# Structural basis of NINJ1-mediated plasma membrane rupture in cell death

**DOI:** 10.1038/s41586-023-05991-z

**Published:** 2023-05-17

**Authors:** Morris Degen, José Carlos Santos, Kristyna Pluhackova, Gonzalo Cebrero, Saray Ramos, Gytis Jankevicius, Ella Hartenian, Undina Guillerm, Stefania A. Mari, Bastian Kohl, Daniel J. Müller, Paul Schanda, Timm Maier, Camilo Perez, Christian Sieben, Petr Broz, Sebastian Hiller

**Affiliations:** 1 Biozentrum, University of Basel, Basel, Switzerland; 2 Department of Immunobiology, University of Lausanne, Epalinges, Switzerland; 3 Stuttgart Center for Simulation Science, Cluster of Excellence EXC 2075, University of Stuttgart, Stuttgart, Germany; 4 Institute of Science and Technology Austria (ISTA), Klosterneuburg, Austria; 5 Department of Biosystems Science and Engineering, Eidgenössische Technische Hochschule (ETH) Zurich, Basel, Switzerland; 6 Nanoscale Infection Biology Group, Department of Cell Biology, Helmholtz Centre for Infection Research, Braunschweig, Germany; 7 Institute for Genetics, Technische Universität Braunschweig, Braunschweig, Germany

**Keywords:** Cryoelectron microscopy, Inflammasome, Apoptosis, Membrane structure and assembly, Super-resolution microscopy

## Abstract

Eukaryotic cells can undergo different forms of programmed cell death, many of which culminate in plasma membrane rupture as the defining terminal event^1–7^. Plasma membrane rupture was long thought to be driven by osmotic pressure, but it has recently been shown to be in many cases an active process, mediated by the protein ninjurin-1^8^ (NINJ1). Here we resolve the structure of NINJ1 and the mechanism by which it ruptures membranes. Super-resolution microscopy reveals that NINJ1 clusters into structurally diverse assemblies in the membranes of dying cells, in particular large, filamentous assemblies with branched morphology. A cryo-electron microscopy structure of NINJ1 filaments shows a tightly packed fence-like array of transmembrane α-helices. Filament directionality and stability is defined by two amphipathic α-helices that interlink adjacent filament subunits. The NINJ1 filament features a hydrophilic side and a hydrophobic side, and molecular dynamics simulations show that it can stably cap membrane edges. The function of the resulting supramolecular arrangement was validated by site-directed mutagenesis. Our data thus suggest that, during lytic cell death, the extracellular α-helices of NINJ1 insert into the plasma membrane to polymerize NINJ1 monomers into amphipathic filaments that rupture the plasma membrane. The membrane protein NINJ1 is therefore an interactive component of the eukaryotic cell membrane that functions as an in-built breaking point in response to activation of cell death.

## Abstract (web summary)

Structural, biochemical and mutagenesis studies indicate that, in dying cells, the membrane protein NINJ1 assembles into filaments, disrupting the cell membrane.

## Main

**Fig. 1:**
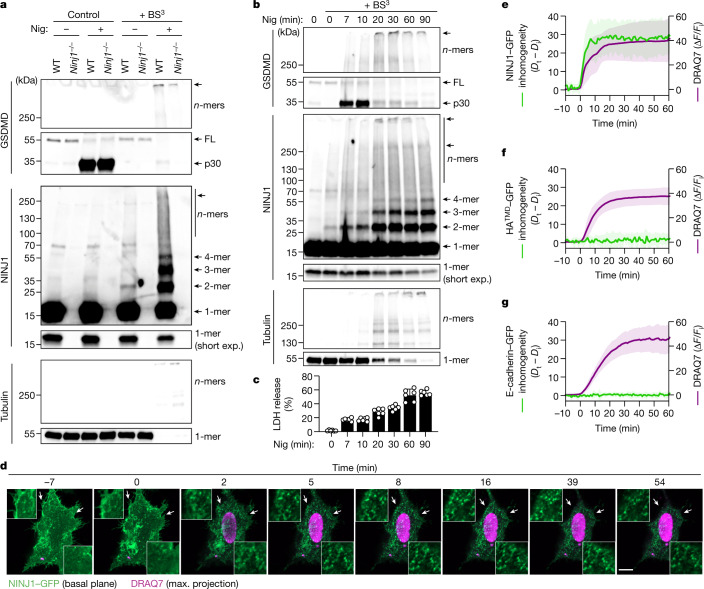
Polymerization kinetics of plasma membrane NINJ1. **a**,**b**, Western blot analysis of endogenous GSDMD and NINJ1 in primed BMDMs after nigericin stimulation (Nig) for 1.5 h (**a**) or for 2, 5, 15, 25, 55 or 85 min (**b**) followed by treatment with the membrane-impermeable BS^3^ crosslinker for 5 min. FL, full length. Of note, tubulin, used here as a loading control, is crosslinked owing to BS^3^ entry through GSDMD pores under nigericin-treated conditions. Time in **b** is the total incubation time for nigericin and BS^3^ treatment. Short exp., short exposure. For gel source data, see Supplementary Fig. 1. **c**, LDH release from primed BMDMs after nigericin stimulation. **d**, Time-lapse fluorescence confocal microscopy of HeLa cells co-expressing hNINJ1–GFP and opto-casp1 following photo-activation. Images show the NINJ1–GFP fluorescence at the basal plane of the cell and the influx of DRAQ7 (maximum (max.) projection from a *z*-stack) to track plasma membrane permeabilization. Time was normalized to the onset of increase in DRAQ7 nuclear fluorescence. White arrows indicate regions that are enlarged in the insets. Scale bar, 10 µm. **e**–**g**, Normalized quantification of the distribution inhomogeneity of NINJ1–GFP (**e**), HA^TMD^–GFP (**f**) or E-cadherin–GFP (**g**) at the basal plane of cells, and DRAQ7 nuclear fluorescence intensity after photoactivation of opto-casp1 (*F*). The distribution inhomogeneity at each time point (*D*_t_) was normalized to the distribution inhomogeneity at the initial time point of the experiment (*D*_i_). Data are mean ± s.d. Data are representative of 2 (**b**), 3 (**a**) or 14 (**d**) independent experiments, or pooled from 2 independent experiments performed in triplicate (**c**) or at least 10 (**e**–**g**) independent experiments. Source data

**Extended Data Fig. 1:**
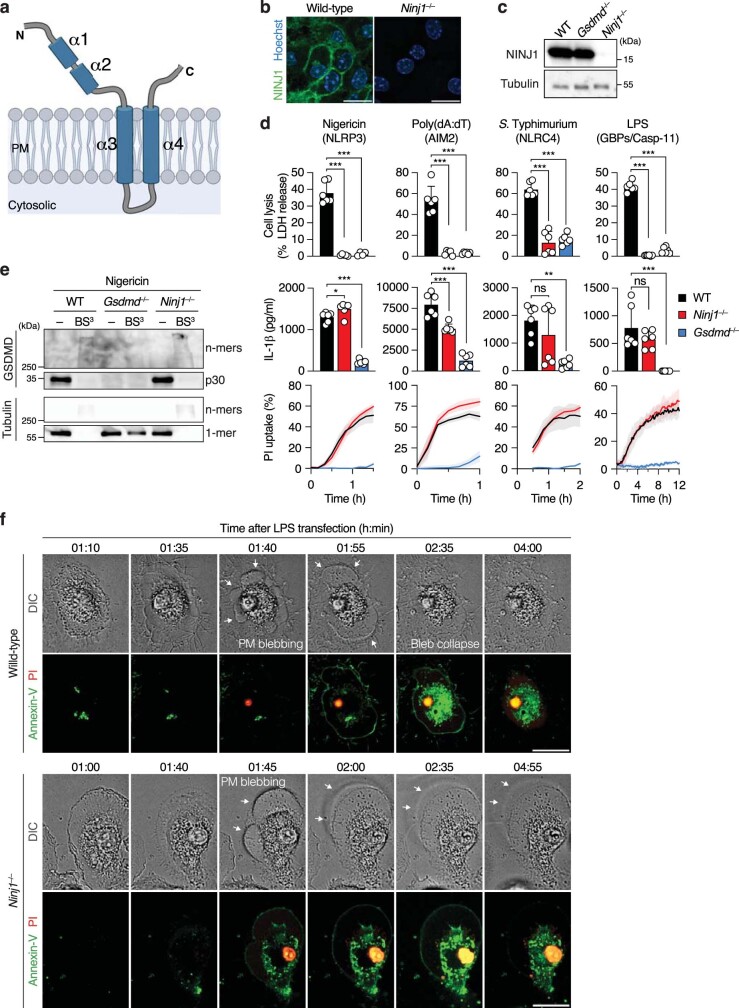
Inflammasome activation induces NINJ1-dependent PMR downstream of GSDMD. **a**, Topology model of non-activated NINJ1 in the plasma membrane (PM)^8^. **b**, Immunofluorescence microscopy of NINJ1 in BMDMs. Scale bars, 15 µm. **c**, Western blot analysis of NINJ1 expression in wild-type (WT), *Gsdmd*^–/–^ and *Ninj1*^–/–^ BMDMs. **d**, Release of LDH or IL-1β in primed WT, *Gsdmd*^–/–^ and *Ninj1*^–/–^ BMDMs stimulated with Nigericin (1 h), transfected with poly(dA:dT) (1 h) or LPS (5 h), or infected with *S*. Typhimurium (1.5 h). Uptake of propidium iodide (PI) was measured every 10 min after the different stimulations. **e**, Western blot analysis of endogenous GSDMD in primed BMDMs after Nigericin stimulation for 1 h followed by treatment with BS^3^ crosslinker for 30 min. **f**, Time-lapse fluorescence microscopy of primed WT and *Ninj1*^–/–^ BMDMs upon transfection with LPS. Pyroptotic cells lost membrane integrity (acquisition of PI, a membrane-impermeable DNA dye) and were labeled by annexin-V (labels phosphatidylserine exposure to the outer leaflet of the plasma membrane). Plasma membrane (PM) blebs observed during pyroptosis (white arrows) collapsed in a NINJ1-dependent manner. DIC, differential interference contrast. Scale bars, 20 µm. Graphs show the mean ± SD and data are representative from 2 (b,c,e,f) or pooled from 2 independent experiments performed in triplicate (d). * *P* < 0.05; ** *P* < 0.01; *** *P* < 0.001; ns, not significant. *P*-values were calculated using one-way ANOVA with multiple comparisons Dunnett tests. For gel source data, see Supplementary Fig. 2. Source data

NINJ1 is a 16-kDa plasma membrane protein that is evolutionarily conserved and found in all higher eukaryotes. It has been predicted to feature two transmembrane helices, with the termini located in the extracellular space^8^ (Extended Data Fig. 1a). During inflammasome-driven pyroptosis, NINJ1 induces plasma membrane rupture (PMR) downstream of the cell death executor gasdermin D (GSDMD), which in turn is activated by caspase-dependent cleavage^8–10^ (Extended Data Fig. 1b–f and Supplementary Videos 1 and 2). PMR coincides with the formation of higher-order NINJ1 polymers and membrane blebs^8^. To better understand the correlation between these events and to study the assembly kinetics of NINJ1 polymers in response to inflammasome activation, we performed crosslinking experiments in mouse bone-marrow-derived macrophages (BMDMs). In line with progressive polymerization of NINJ1, we detected the formation of NINJ1 dimers and trimers 10 min after inflammasome activation; this was followed by extensive polymerization of NINJ1 and the formation of larger polymers at later time points (Fig. 1a,b). The bulk of NINJ1 polymerization coincided with complete oligomerization of cleaved GSDMD p30 (Fig. 1a,b)—that is, formation of GSDMD pores—consistent with NINJ1 activation occurring downstream of GSDMD activation. PMR was quantified by the release of lactate dehydrogenase (LDH), which is too large to be released directly through GSDMD pores^11–13^. Of note, following inflammasome activation, the amount of released LDH increased slowly with time, whereas NINJ1 polymerization was already detectable at the onset of LDH release and increased only marginally at later time points (Fig. 1c). Small amounts of NINJ1 dimers were also detectable in live cells (Fig. 1a,b), suggesting that higher-order NINJ1 polymers are the active species. These time-resolved data are thus fully consistent with a necessity of NINJ1 polymerization for PMR.

**Extended Data Fig. 2:**
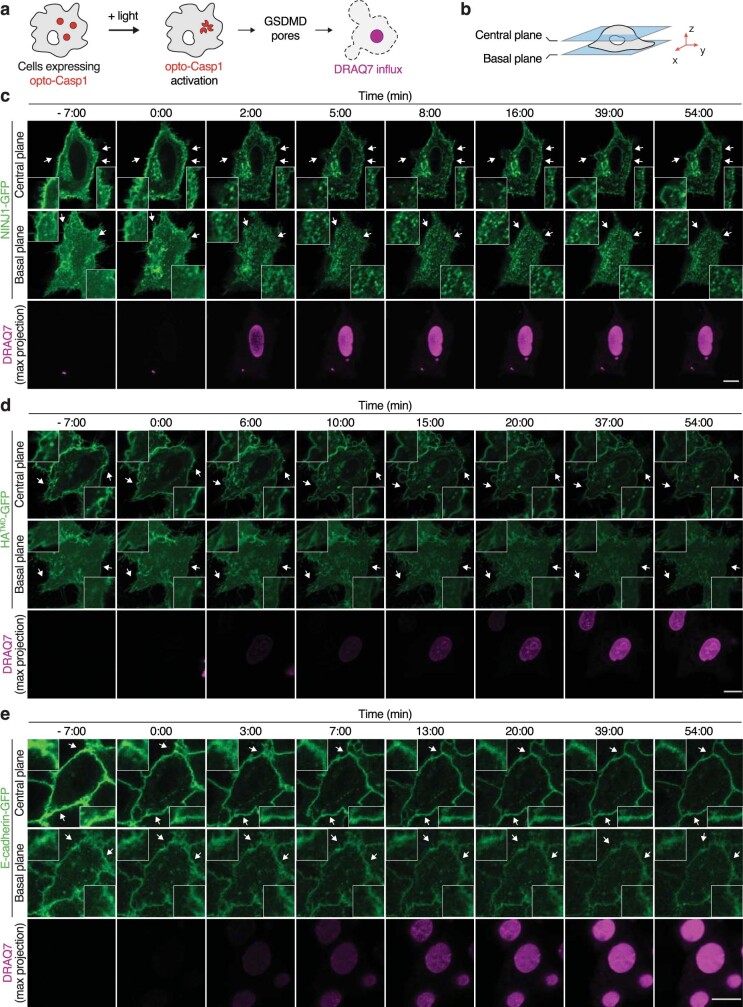
NINJ1 quickly polymerizes at the plasma membrane upon pyroptosis activation. **a**,**b**, Schematic representation of the experimental setup used to analyze NINJ1 polymerization at the plasma membrane upon optogenetic induction of GSDMD-driven pyroptosis in single cells: HeLa cells expressing Opto-Casp1 (red dots) are photo-activated with 488 nm light to induce rapid Opto-Casp1 clustering and caspase activation, triggering GSDMD pore formation and influx of DRAQ7 (a membrane-impermeable DNA dye) (a). Time-lapse fluorescence confocal microscopy allows to follow NINJ1-GFP localization and clustering in different Z-planes of the cell (b). **c**—**e**, Time-lapse fluorescence confocal microscopy images (basal or central plane of cells) of HeLa cells co-expressing Opto-Casp1 and hNINJ1-GFP (c), HA^TMD^-GFP (d) or E-cadherin-GFP (e), after photo-activation. DRAQ7 influx (maximum projection from a Z-stack) shows plasma membrane permeabilization during cell death. Time was normalized to the onset of increase in DRAQ7 nuclear fluorescence. Scale bars correspond to 10 µm and white arrows point to inset images. Data are representative from fourteen (c), eleven (d) or ten (e) independent experiments.

**Extended Data Fig. 3:**
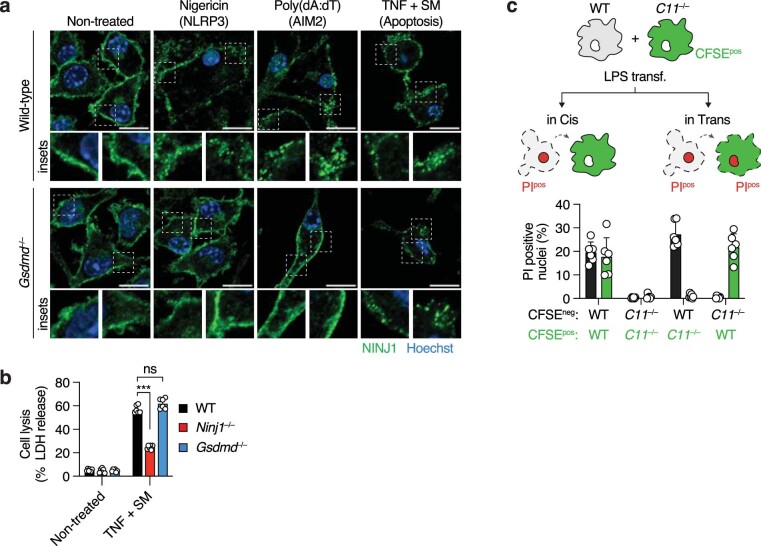
Cell death triggers NINJ1 polymerization at the plasma membrane and induces PMR in a cell-intrinsic manner. **a**, Immunofluorescence microscopy of endogenous NINJ1 in primed BMDMs upon stimulation with Nigericin (1 h), or poly(dA:dT) transfection (1 h) (to induce pyroptosis), or in naïve cells stimulated with TNF + SM (SMAC-mimetic AZD 5582) for 16 h (to induce apoptosis). Scale bars, 10 µm. **b**, LDH release in WT, *Gsdmd*^–/–^ or *Ninj1*^–/–^ BMDMs stimulated with TNF + SM (16 h). **c**, Schematic representation of the experimental setup to assess if NINJ1-driven PMR acts in a cell-intrinsic (in Cis) or -extrinsic (in Trans) manner: primed WT BMDMs were co-cultured with *Casp11*^–/–^ (*C11*^–/–^) BMDMs previously incubated with CFSE. Co-cultured cells were then transfected with purified LPS and the incorporation of propidium iodide (PI) in dying cells (dashed contour) was quantified in both cell types by fluorescence microscopy. Different co-cultures were prepared (WT + WT^CFSE-pos^; *C11*^–/–^ + *C11*^–/–CFSE-pos^; WT + *C11*^–/–CFSE-pos^; *C11*^–/–^ + WT^CFSE-pos^), and the percentage of cells with PI positive nuclei was quantified in the CFSE-negative and -positive cells. Graphs show the mean ± SD and data are representative from two independent experiments (a), or pooled from two independent experiments performed in triplicate (b,c). *** *P* < 0.001; ns, not significant. *P*-values were calculated using one-way ANOVA with multiple comparisons Dunnett tests. Source data

We also monitored NINJ1 polymerization using time-lapse fluorescence microscopy in HeLa cells co-expressing GFP-tagged human NINJ1 (hNINJ1) and a CRY2–caspase-1 fusion protein (opto-casp1), which enables rapid caspase activation and induction of GSDMD-driven pyroptosis in single cells using optogenetics^14^ (Fig. 1d and Extended Data Fig. 2a–c). Quantitative inhomogeneity analysis showed that concurring with the influx of the dye DRAQ7, which indicates a loss of plasma membrane integrity and cell death, the diffusely localized NINJ1 started to cluster at the plasma membrane (Fig. 1d,e, Extended Data Fig. 2c and Supplementary Video 3), and that these clusters persisted beyond cell lysis. Notably, the formation of clusters during pyroptosis was specific for NINJ1, as other plasma membrane proteins such as the haemagglutinin transmembrane domain^15^ (HA^TMD^) or E-cadherin did not cluster (Fig. 1f,g, Extended Data Fig. 2d,e and Supplementary Videos 4 and 5). Similar assemblies were also formed by endogenous NINJ1 in wild-type but not in *Gsdmd*^—/—^ BMDMs upon activation of the NLRP3 or AIM2 inflammasomes (Extended Data Fig. 3a). Clustering of NINJ1 and NINJ1-dependent LDH release was also detected upon induction of apoptotic cell death^8^ (Extended Data Fig. 3a,b), but in this case independently of GSDMD. Next, we investigated whether NINJ1-driven PMR is a cell-intrinsic process or whether this activity is dependent on neighbouring cells via released NINJ1 or direct contact. Co-culture experiments of wild-type BMDMs with *Casp11*-deficient BMDMs transfected with lipopolysaccharide (LPS) to activate the non-canonical inflammasome unambiguously demonstrated that NINJ1 lyses cells in a cell-intrinsic manner without affecting immediate neighbours (Extended Data Fig. 3c). NINJ1-driven PMR in inflammasome-activated cells is thus a cell-intrinsic process, which involves the formation of GSDMD pores and rapid NINJ1 polymerization, followed by membrane rupture with slower kinetics.

## NINJ1 polymerizes into filaments

**Fig. 2:**
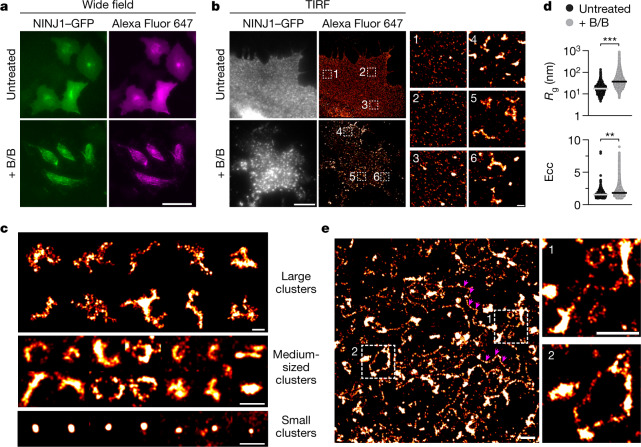
Super-resolution imaging of NINJ1 assemblies. **a**, Wide-field imaging of DmrB–Casp4^tg^ HeLa cells expressing hNINJ1–GFP and GSDMD used for STORM microscopy in **b**–**e**. Cells were left untreated or stimulated with B/B homodimerizer 3 h before fixation and labelling with Alexa Fluor 647-conjugated anti-GFP nanobodies. Scale bar, 50 µm. **b**–**e**, STORM super-resolution imaging of hNINJ1–GFP in cells from **a** using TIRF illumination of the basal plane. **b**, Left, untreated or B/B-stimulated cells expressing hNINJ1–GFP. Scale bar, 10 µm. **b**, Right, STORM super-resolution reconstruction of hNINJ1–GFP labelled with Alexa Fluor 647-conjugated anti-GFP nanobodies. The indicated outlined regions are magnified on the right. Scale bar, 500 nm. **c**, Gallery of hNINJ1–GFP clusters found in pyroptotic cells. The small clusters are also observed in non-activated cells. Scale bars, 500 nm. **d**, Radius of gyration (*R*_g_) and eccentricity (Ecc) for each identified hNINJ1 cluster. Plots show the distribution of all identified clusters from three independent experiments. The lines indicate median values. Statistical analysis based on the median *R*_g_ and Ecc of each experiment using Student’s unpaired two-sided *t*-test. **P < 0.01, ***P < 0.001. **e**, Overview STORM reconstruction of assemblies in B/B-stimulated cells expressing hNINJ1–GFP including filamentous structures. Two filaments are highlighted with magenta arrowheads. The indicated regions are magnified on the right. Scale bars, 1 µm. Data in **a**–**c**,**e** are representative of at least three independent experiments. Source data

**Extended Data Fig. 4:**
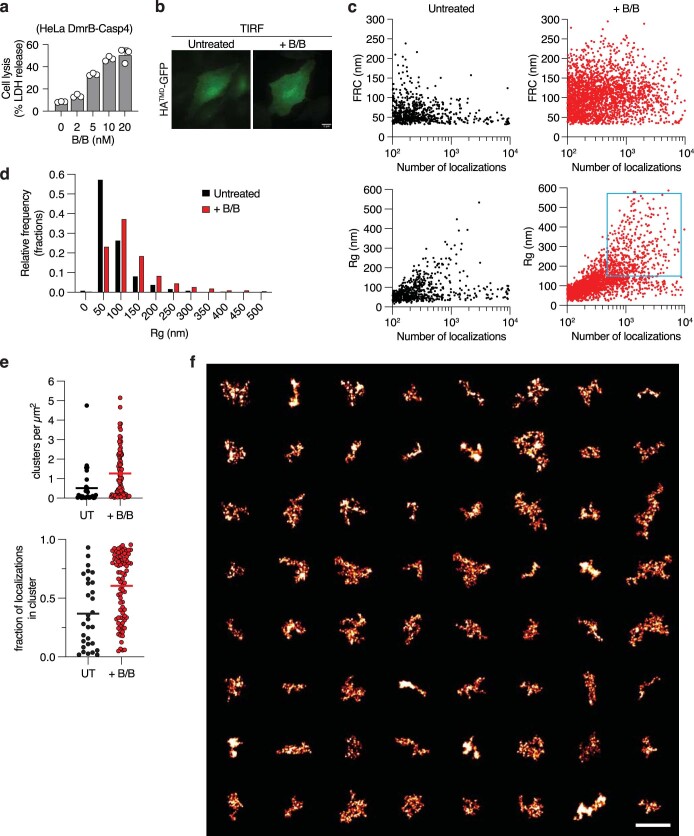
NINJ1-GFP cluster size and resolution after density-based clustering. **a**, Release of LDH in HeLa cells expressing DmrB-Casp4 upon activation with different doses of B/B homodimerizer for 3 h. Graph shows the mean ± SD and data are representative from two independent experiments performed in triplicate. **b**, Absence of reorganization of the control protein HA^TMD^-GFP upon cell death. HeLa cells co-expressing DmrB-Casp4 and HA^TMD^-GFP^15^ were untreated (UT) or stimulated with B/B homodimerizer and imaged using TIRF microscopy to visualize the membrane organization of HA^TMD^-GFP. **c**, Density-based clustering of NINJ1 clusters, correlating their FRC resolution and size (Rg). Clustering was performed in multiple regions of interest (ROIs) across multiple cells, with equal total area for each condition. Rg and FRC are plotted against the number of localizations per cluster for each identified cluster. For plotting and further analysis in panels **d**–**f**, only clusters with more than 100 localizations were used. **d**, Histogram of cluster sizes (Rg). **e**, Distribution of cluster per area cell surface. Analysed were 23/72 cells from 2/4 independent experiments (UT/+BB). Lines indicate mean values. **f**, Extended gallery of large NINJ1-GFP structures found in pyroptotic cells. Gallery of clusters identified after stimulation (as in Fig. 2c) and selected by the cyan quadrant (Rg > 200 nm, localizations > 500) in **c**. Scale bar, 2 µm. Source data

We next performed stochastic optical reconstruction microscopy (STORM) to investigate the nanoscale organization of NINJ1 assemblies in pyroptotic cells. To this end, we used HeLa cells co-expressing hNINJ1–GFP and DmrB–caspase-4. DmrB is a dimerization module that enables activation of caspase-4, the human caspase-11 orthologue, and subsequent activation of GSDMD^16,17^ by an exogenous, cell-permeable ligand^18^ (Extended Data Fig. 4a). We used an anti-GFP nanobody and wide-field epi-fluorescence imaging to specifically label and image hNINJ1–GFP structures (Fig. 2a). For STORM, total internal reflection fluorescence (TIRF) illumination with single-molecule localization analysis was used to super-resolve NINJ1 assemblies in the plasma membrane (Fig. 2b). Live cells showed a homogeneous distribution of small NINJ1 dots, which could be individual molecules or small polymers, and a few small and mostly round clusters (Fig. 2b). By contrast, cells undergoing pyroptosis displayed larger NINJ1 clusters, with dimensions ranging from about 500 nm to several micrometres (Fig. 2b,c). The morphology of these clusters was highly branched, with individual branches protruding in different directions, somewhat resembling the organization of the filamentous ASC speck. These clusters correspond to the large dots observed by wide-field epi-fluorescence and confocal microscopy (Fig. 1d and Extended Data Fig. 2c). Again, cluster formation during pyroptosis was not observed for the control protein HA^TMD^ (Extended Data Fig. 4b). To quantify the change in NINJ1 nanoscale organization upon activation of cell death, we performed density-based clustering to determine the number of clusters per area, the distributions of cluster sizes (radius of gyration (*R*_g_)) and shapes (eccentricity (Ecc)), and the number of single-molecule localizations per NINJ1 cluster. This analysis showed that after caspase activation, more clusters were identified per cell surface area and the clusters were significantly larger and less spherical than in non-activated cells (Fig. 2d and Extended Data Fig. 4c–f). In around 10% of cells, we also observed long NINJ1 filaments up to several micrometres in length that connected the larger assemblies (Fig. 2c,e). In summary, super-resolution microscopy analysis showed that in pyroptotic cells, NINJ1 polymerizes into large clusters with various shapes of branched, filamentous morphology, as well as long filaments in the micrometre range.

## Atomic structure of the NINJ1 filament

**Fig. 3:**
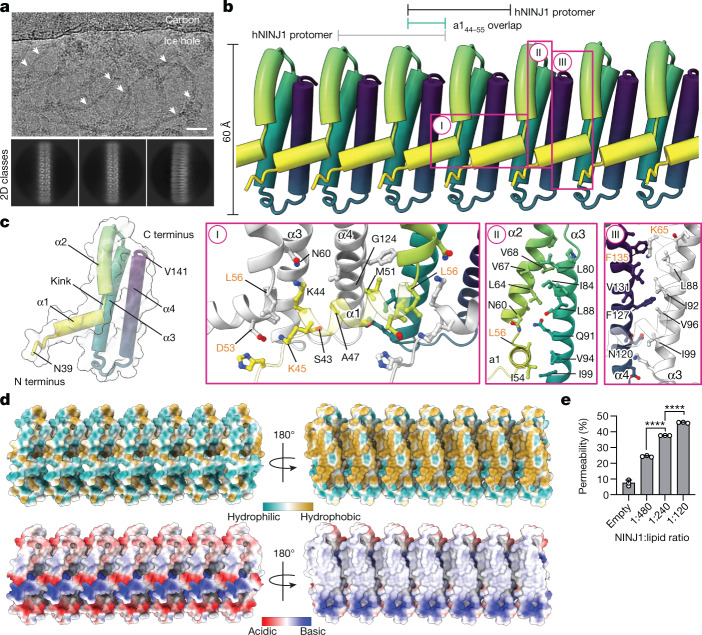
Cryo-EM structure of NINJ1 filament. **a**, Cryo-EM micrograph showing filamentous hNINJ1 (white arrows) along representative 2D classes. Scale bar, 25 nm. Micrograph representative of 13,124 micrographs from one dataset. **b**, Organization of hNINJ1 filaments with helices represented as tubes and each subunit shown in a colour gradient (yellow–green–purple). The main interaction interfaces I, II and III are shown below. **c**, A single hNINJ1 filament subunit, comprising helices α1–α4, with surface representation outlined in light grey. **d**, Lipophilicity and charge distribution of the hNINJ1 filament. **e**, Permeability of hNINJ1 proteoliposomes at different protein:lipid molar ratios. Data are mean + s.d. (*n* = 3 independent experiments). Statistical analysis by one-way ANOVA. ****P < 0.0001. Source data

**Extended Data Fig. 5:**
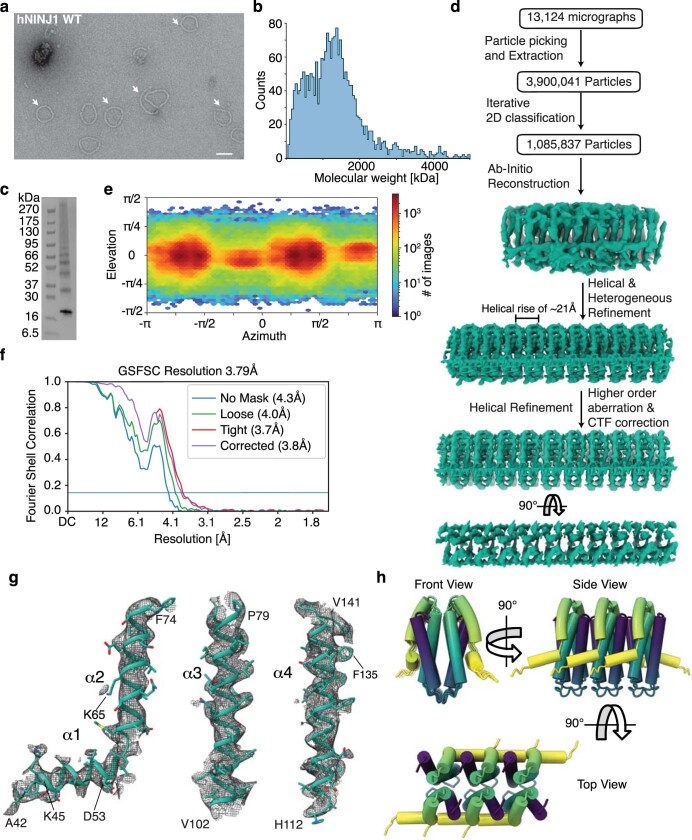
Structure determination of hNINJ1 filaments. **a**, Negative Stain TEM micrograph of purified hNINJ1 ring assemblies (white arrows). Scale bar, 50 nm. Micrograph representative of *n* = 14 independent experiments. **b**, Mass photometry measurements of purified hNINJ1 in presence of detergent. **c**, Western Blot of purified hNINJ1 sample used for cryo-EM structure determination. For gel source data, see Supplementary Fig. 3. **d**, Cryo-EM processing workflow. **e**, Distribution of orientations over azimuth and elevation angles for particles included in the calculation of the final map (*n* = 709,840 particles). **f**, Gold standard Fourier shell correlation (GSFSC) plot for final helical refinement of hNINJ1 dataset. **g**, Modeled helices α1−α4 shown individually within the experimentally determined cryo-EM density (grey mesh). **h**, Collection of views of the hNINJ1 double filament model.

**Extended Data Fig. 6:**
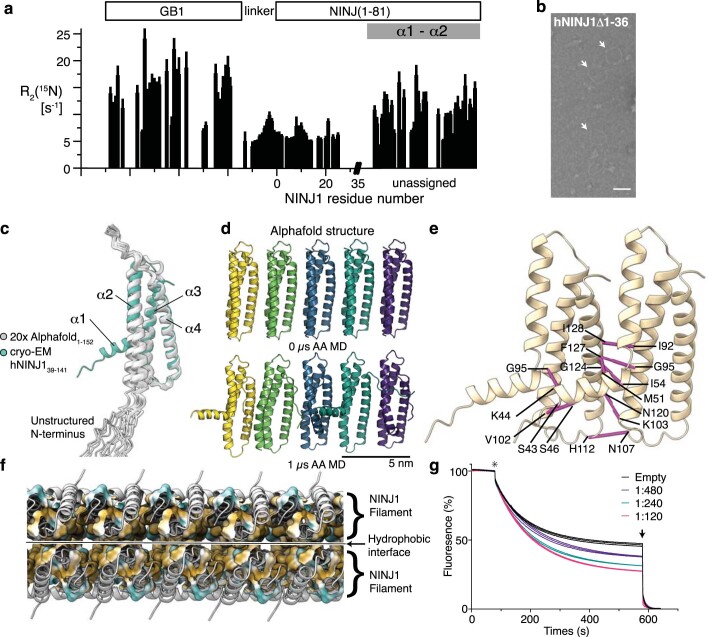
Structural and functional analysis of NINJ1. **a**, ^15^N R_2_ transverse relaxation rate measurements of GB1-hNINJ_1–82_ in the absence of any membrane-mimicking environment show that the N-terminal ~40 residues are highly flexible, while the remainder has limited flexibility. **b**, Negative stain micrograph of purified hNINJ1∆1–36 showing ring assemblies (white arrows), scale bar corresponds to 50 nm. Micrograph representative of *n* = 2 independent experiments. **c**, Overlay of 20 Alphafold predictions of hNINJ1 with the experimentally determined structure. **d**, Snapshots at 0 µs and 1 µs of an AA simulation of a hypothetical filament, where the experimental monomer was replaced by the Alphafold-predicted monomer (n = 1). The backbone of helices α3 and α4 was restrained to study the propagation of helices α1 and α2. **e**, Residue pairs with co-evolutionary coupling displayed on the filament structure of hNINJ1. Shown are all pairs among the 100 most significant couplings, that are closer in space in the filament than in the monomer. A list of these pairs is given in Supplementary Table 2. **f**, Hydrophobic interaction occurring between hNINJ1 filaments. **g**, Relative fluorescence traces of hNINJ1 proteoliposomes with different lipid to protein molar ratios, as indicated. Star indicates the addition of dithionite. Arrow indicates timepoint of Triton X-100 addition. Triplicates are shown. Source data

To characterize polymeric NINJ1 at the atomic level, we expressed full-length, wild-type hNINJ1 (residues 1–152) recombinantly in *Escherichia coli* and extracted it from the bacterial membrane with the detergent *n*-dodecyl-β-d-maltopyranoside (DDM). The purified protein was visualized by negative staining and cryo-electron microscopy (cryo-EM), showing long filaments with different degrees of bending that occasionally closed to form rings (Fig. 3a and Extended Data Fig. 5a). Quantification of the mass of purified hNINJ1 filaments indicated an average molecular mass of around 1.3 MDa (Extended Data Fig. 5b). The structure of hNINJ1 filaments was determined by cryo-EM. Well-resolved 2D classes representing a top view and initial 3D volumes were used to determine helical parameters via a power spectrum analysis and subsequent helical refinement yielded a final cryo-EM map (Extended Data Fig. 5c–h). The filaments were linear stacks of subunits, with an interval of 20.95 Å and a slight rotation of –1.05° per subunit. The cryo-EM map had a resolution of 3.8 Å, enabling us to build a molecular model of filamentous hNINJ1 (Fig. 3a,b and Extended Data Table 1). The first 38 residues of hNINJ1 remained disordered, as shown by solution NMR relaxation experiments (Extended Data Fig. 6a), in full agreement with predictions from AlphaFold and previous NMR experiments^19,20^. Indeed, a truncation experiment showed these residues to be dispensable for hNINJ1 filament formation (Extended Data Fig. 6b). The next 103 residues (residues 39–141) were well represented in the maps and could be modelled unambiguously as four α-helices α1–α4 (Extended Data Fig. 5g). Notably, the experimental density comprised two identical hNINJ1 filaments, which were packed together in an antiparallel arrangement (Extended Data Fig. 5h).

The hNINJ1 filament is organized by stacking of adjacent protomers in a fence-like manner (Fig. 3b). The two antiparallel helices α3 and α4 (residues 79–103 and 114–138, respectively) form the core of the filament. These helices are hydrophobic and form a hairpin of transmembrane helices in the inactive form of the protein (Extended Data Fig. 1a). The two N-terminal helices α1 and α2 (residues 44–55 and 58–74, respectively) are separated by a distinct kink at L56 (Fig. 3c). α2 thus adopts a parallel orientation with respect to α3 and α4, whereas α1 protrudes at a nearly 90° angle from the helical bundle and connects to the adjacent protomer via an extensive polymerization interface. The intermolecular contacts include multiple side chain interactions between helix α1 of one protomer and helices α1, α3 and α4 of the neighbouring protomer, of which a salt bridge between the highly conserved residues K45 and D53 is the most prominent (Fig. 3b, I). The interaction also includes newly formed intramolecular contacts via an extensive hydrophobic patch on the amphipathic helix α2 that matches a complementary side chain array on α3 (Fig. 3b, II). Finally, hydrophobic residues on α3 align with a complementary set of residues on α4 of the neighbouring protomer, presumably including a cation–π interaction between K65 and F135 (Fig. 3b, III).

Notably, the experimental structure of NINJ1 in the filament overlaps nearly perfectly with the AlphaFold model for helices α2, α3 and α4, but not for helix α1 (Extended Data Fig. 6c). In the AlphaFold model, α1 and α2 combine to form a single, straight helix. We used molecular dynamics simulations of hypothetical filaments, in which we replaced the individual monomers in the experimentally determined structure with the AlphaFold model. In several subunits, the single α1–α2 helix began to kink at residue 56 and helix α1 restructured itself towards the cryo-EM structure (Extended Data Fig. 6d). Furthermore, we analysed co-evolution of NINJ1, which also underlies the model building in AlphaFold, and focused on the 100 most significant co-evolution pairs. A large majority of these residues pairs corresponds to intramolecular short-range contacts within the NINJ1 monomer. Nine of the residue pairs, however, are in closer spatial proximity between subunits in the filament structure than within one monomer (Extended Data Fig. 6e). In particular, this includes the residue pair F127–G95, which has the highest significance score of all pairs in NINJ1 and which intermolecularly connects helices α3 and α4. These evolutionary data lend further support for the relevance of filamentous NINJ1 in a biological context.

## NINJ1 filaments rupture membranes

When analysing the surface hydrophobicity of the NINJ1 filament, we observed that one face of the filament is hydrophilic whereas the other face is hydrophobic (Fig. 3d). These properties directly explain how two hNINJ1 filaments have stacked via their hydrophobic faces to result in the soluble double filament resolved by cryo-EM (Extended Data Fig. 6f). The topology of the filament with one hydrophobic and one hydrophilic face is typical for pore-forming proteins such as gasdermins, perforin or bacterial toxins^13,21–24^, and thus readily connects to a functional role in the cell, where these filaments probably cap membrane edges, enabling the rupture of a lipid bilayer membrane. Consistent with this notion, the curved hydrophobic interface measures around 26 Å in height, matching the average thickness of the eukaryotic plasma membrane^25^. We tested the ability of NINJ1 to increase membrane permeability in liposomes. We reconstituted NINJ1 into proteoliposomes comprising 1-palmitoyl-2-oleoyl-*sn*-glycero-3-phosphoethanolamine (POPE) and 1-palmitoyl-2-oleoyl-*sn*-glycero-3-phospho-(1′-rac-glycerol) (POPG) lipids, together with traces of the fluorescent lipid nitrobenzoxadiazole-phosphatodylcholine (NBD-PC). Addition of dithionite, a membrane-impermeable molecule, quenches the fluorescence of NBD-PC only on the leaflets accessible to the bulk solution. Consistent with the hypothesis that NINJ1 induces membrane rupture, the relative permeability of NINJ1 proteoliposomes increased with the protein:lipid ratio of hNINJ1 in the membrane (Fig. 3e and Extended Data Fig. 6g).

**Extended Data Fig. 7:**
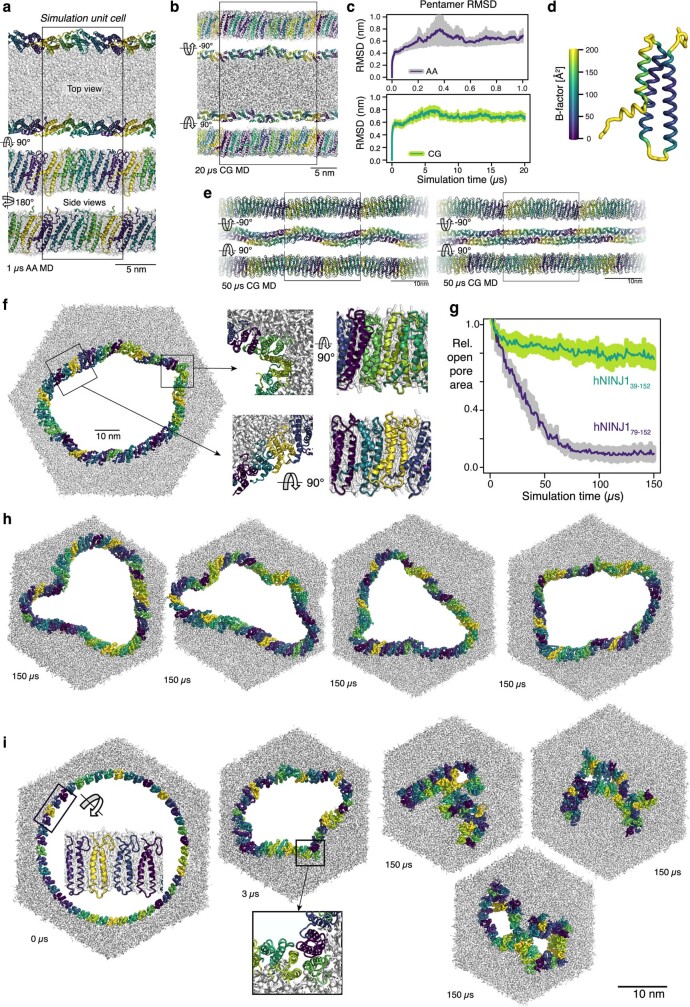
Stability of NINJ1 polymers probed by molecular dynamics simulation. **a**, Snapshot after 1 µs of an all-atom simulation of linear NINJ1 oligomers on both edges of a membrane stripe (n = 2). **b**, Snapshot after 20 µs of a CG MD simulation of linear filaments capping the edges of a membrane stripe (n = 2). **c**, Root mean square deviations (RMSD) over simulation time of the backbone of linear pentamers attached to the membrane patches. Average RMSD and SEM over individual, unique pentamers and simulations (n = 4 for AA and n = 8 for CG) are given. **d**, Average structure and B-factors (in Å^2^) of the hNINJ1_39–152_ backbone (n = 20) in simulations (a). Structures were aligned to helices α3 and α4 prior analysis. **e**, Representative snapshots of CG MD simulations of the hNINJ1_39–152_ double filament after 50 µs in absence (left, n = 2) and presence (right, n = 2) of DDM. The black box highlights the simulation unit cell. Periodic filament copies were added on right and left. **f**, Representative snapshot after 40 µs of a CG simulation of a circular hNINJ1_39–152_ 45-mer in a lipid bilayer (n = 4). Insets on the right show the protein packing in a straight (bottom) and in a curved part of the polymer (top). **g**, Time evolution of the average inner area of rings made of 45 hNINJ1_39–152_ (n = 4) or hNINJ1_76–152_ (n = 3) relative to the area at *t* = 3 µs. The shaded areas show SEM over individual simulations. **h**, Snapshots at 150 µs of four independent CG MD simulations of 45-mer hNINJ1_39-152_ (n = 4). **i**, Starting structure, collapse at 3 µs, as well as three snapshots at 150 µs of independent CG MD simulations of 45-mer hNINJ1_76–152_ rings, i.e. missing helices α1 and α2 (n = 3). Lipids are shown as grey sticks, with phosphates highlighted as spheres. hNINJ1 protomers are randomly colored yellow, green or purple with the exception of **c**, where the purple-green-yellow coloring scales with the B-factor (purple low B-factor, yellow large B-factor). Source data

Next, we used molecular dynamics simulations to explore the stability of ultrastructural hNINJ1 organizations at membrane edges in silico. Two linear filaments on the opposite edges of a membrane patch remained compact and stable for at least 20 µs in coarse-grained simulations, and for at least 1 µs in all-atom simulations—this was confirmed in two independent replicates each and in control simulations with the experimental double filament (Extended Data Fig. 7a–e). Next, we created a small NINJ1 pore in a membrane in silico, by rearranging a 45-mer filament into a ring. In four coarse-grained simulations of 150 µs length, the NINJ1 polymer remained structurally stable, while showing some variability in the relative orientation of the neighbouring protomers (Extended Data Fig. 7f–h and Supplementary Video 6). In stark contrast, analogous simulations of a ring of truncated NINJ1 lacking helices α1 and α2 underwent a structural collapse and closure of the ring polymers (Extended Data Fig. 7i and Supplementary Video 7). Within a few microseconds, interactions formed between helices α3 and α4 of nearby protomers, and these propagated within tens of microseconds throughout the whole polymer, almost completely closing the ring. Since under similar simulation conditions and duration, structurally defective gasdermin A3 pores undergo substantial reshaping^26,27^, the observation of stable NINJ1 pores and the collapse of a truncated variant strongly suggest that NINJ1 filaments can cap membrane edges in variable arrangements. Helix α1 thus both stabilizes NINJ1 filaments and confers local plasticity, which could be crucial when perforating densely packed cellular membranes.

## Functional validation in cells

**Fig. 4:**
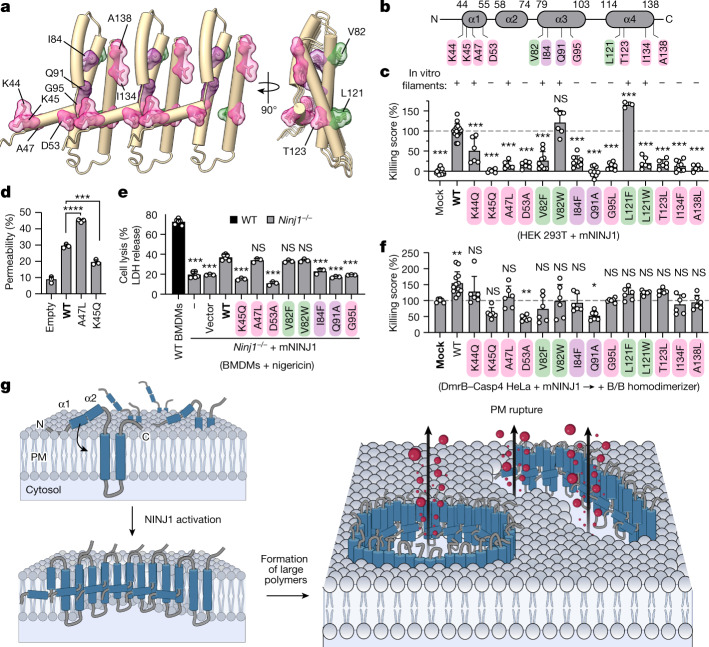
The mechanism of NINJ1-mediated PMR. **a**, Three subunits of filamentous hNINJ1 with overview of residues selected for mutagenesis study (intermolecular interactions, magenta; intramolecular interaction, purple; membrane interactions, green). **b**, Schematic representation of the residues selected for mutagenesis. **c**, Cytotoxicity upon overexpression of wild-type (WT) or mutant mNINJ1 in HEK 293T cells. **d**, Permeability of proteoliposomes containing wild-type and mutant mNINJ1 compared with protein-free liposomes (empty). Data are mean + s.d. (*n* = 3). **e**, Release of LDH in primary *Ninj1*^–/–^ BMDMs reconstituted with wild-type mNINJ1 or different mNINJ1 mutants upon nigericin treatment (1.5 h). Reconstitution with the empty vector and non-transduced *Ninj1*^–/–^ BMDMs (–) were used as controls. **f**, Cytotoxicity upon B/B treatment in HeLa cells co-expressing DmrB–caspase-4 and wild-type or mutant mNINJ1. Killing score corresponds to the cytotoxicity, measured by LDH release, normalized against wild-type mNINJ1 control (**c**) or mock-treated controls (**f**). Statistical analysis in **c**–**f** by individual comparison to the control condition highlighted in bold. Data are mean + s.d. and data are pooled from two independent experiments performed in triplicate (**c**; for K45Q, K45Q, A47L, D53A, V82W, L121W, T123L and A138L mutants), three independent experiments performed in triplicate (**c**; for mock, WT and V82F, I84F, Q91A, G95L and A138L mutants), and representative of two independent experiments performed in triplicate (**c**; for L121F mutant), pooled from two independent experiments performed in triplicate (**f**) or representative of two independent experiments performed in triplicate (**e**). In **c**,**e**,**f**, multiple plates were used to test all mutants, thus control conditions were included in each of the plates. *P < 0.05, **P < 0.01, ***P < 0.001, **** *P* < 0.0001; NS, not significant. *P* values by one-way ANOVA with Dunnet’s multiple comparisons tests. **g**, Structural model of NINJ1-mediated membrane rupture. Non-activated NINJ1 is randomly distributed in the plasma membrane (PM). Upon activation, NINJ1 polymers lyse the membrane, resulting in the release of cytosolic content (red). Source data

**Extended Data Fig. 8:**
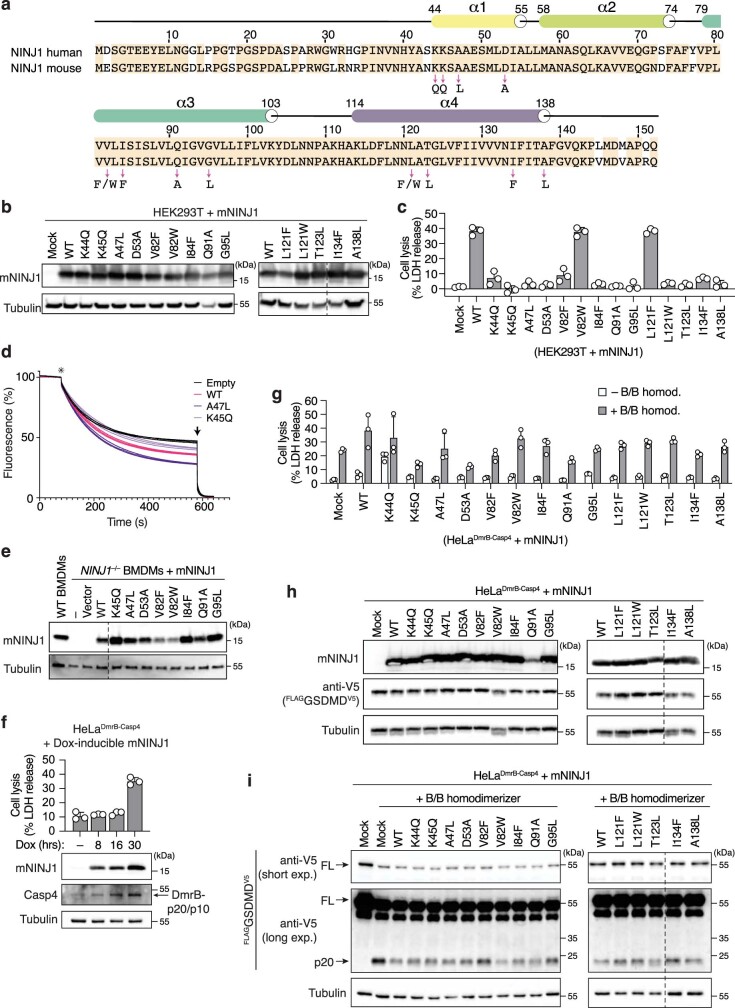
Analysis of NINJ1 single-point mutants. **a**, Comparison of hNINJ1 and mNINJ1 sequences. Orange background shows identical residues. Secondary structure is indicated on top. In the structured part (residues 44–138), 2 amino acids differ and 93 are identical. Arrows indicate selected single residues that were mutated in this work. **b**, **c**, Western blot analysis of mNINJ1 expression (b) and LDH release (c) in HEK 293T cells transiently transfected with WT or different mNINJ1 mutants. mNINJ1 expression was induced by adding doxycycline (Dox) for 48 h. **d**, Triplicates of fluorescence traces of WT-NINJ1, mutants, and empty liposomes. Star indicates the addition of dithionite. Arrow indicates timepoint of Triton X-100 addition. **e**, Western blot analysis of mNINJ1 expression in *Ninj1*^–/–^ primary BMDMs transduced with a retroviral vector expressing WT or different mNINJ1 mutants. Transduction with the empty vector or non-transduced *Ninj1*^–/–^ cells (–) were used as controls. **f**, LDH release and western blot analysis of mNINJ1 expression in HeLa stably expressing FLAG-GSDMD-V5 and Dox-inducible DmrB-Casp4, upon transient transfection with Dox-inducible mNINJ1 and induction of protein expression for the indicated time points. **g**, LDH release in HeLa cells co-expressing Dox-inducible DmrB-Casp4 and Dox-inducible WT or different mNINJ1 mutants. Cells were treated with Dox for 16 h, followed by treatment with B/B homodimerizer. B/B untreated cells were also used as control. **h**, Western blot analysis of mNINJ1 and GSDMD expression in HeLa cells stably co-expressing FLAG-GSDMD-V5 and Dox-inducible DmrB-Casp4, and transiently expressing WT or different mNINJ1 mutants. **i**, Western blot analysis of GSDMD processing in HeLa cells stably co-expressing FLAG-GSDMD-V5 and Dox-inducible DmrB-Casp4, and transiently expressing WT or different mNINJ1 mutants, upon caspase-4 activation with B/B homodimerizer. Cell lysates and supernatants were combined and analyzed. In (h,i) mNINJ1 expression was induced with Dox for 16 h. Graphs show the mean ± SD and data are representative from at least two independent experiments performed in triplicate. For gel source data, see Supplementary Fig. 4. Source data

To validate our structural model, we designed 14 single-amino-acid mutants and tested their effects on hNINJ1 filament formation in vitro and on cell lysis upon overexpression of mouse NINJ1 (mNINJ1) in cells (Fig. 4a–c). Eight mutants at intermolecular interfaces (K44Q, K45Q, A47L, D53A, G95L, T123L, I134F and A138L) and two mutants at intramolecular interfaces (I84F and Q91A) were designed to potentially break the filament structure, and four mutants at the hydrophobic interface to the membrane (V82F, V82W, L121F and L121W) were designed to be compatible with NINJ1 polymerization. Human and mouse NINJ1 are 98% identical in the structured region of residues 44–138, which includes all 12 mutated sites (Extended Data Fig. 8a). Eight of the ten mutants designed to disrupt filament formation (K45Q, D53A, I84F, Q91A, G95L, T123L, I134F and A138L) reduced or completely abrogated cell lysis upon overexpression in HEK 293T cells, and consistently showed reduced filament formation in vitro (Fig. 4c and Extended Data Fig. 8b,c). Among these, the previously reported K45Q mutant^8^ showed significantly decreased membrane permeability compared with the wild type in the liposome permeability assay (Fig. 4d and Extended Data Fig. 8d). Mutant A47L was non-functional in HEK 293T cells, but still formed filaments in vitro and increased permeability of proteoliposomes, suggesting that it is involved in additional interactions in cells. Mutant K44Q showed a highly variable phenotype, preventing interpretation. The four mutations in the hydrophobic interface maintaining its hydrophobicity (V82W, V82F, L121F and L121W) did not affect filament formation in vitro as expected (Fig. 4c). Upon overexpression in cells, V82W and L121F were fully functional, whereas mutations V82F and L121W induced lower levels of cell lysis than wild-type NINJ1. This outcome showed that the two sites can tolerate some, but not necessarily all mutations that maintain the hydrophobicity, in full agreement with the structural model. Furthermore, to test the effect of mutating the interaction interfaces between NINJ1 protomers on inflammasome-induced pyroptosis in a physiological setting, we transduced primary *Ninj1*^−/−^ mouse BMDMs with retroviral vectors expressing either wild-type mNINJ1 or mutants designed to disrupt filament formation and treated the cells with nigericin to activate the NLRP3 inflammasome (Fig. 4e and Extended Data Fig. 8e). In line with the in vitro and HEK 293T overexpression studies, we found that NINJ1 mutants abrogated nigericin-induced release of LDH in reconstituted BMDMs. The sole exception was the A47L mutant, which was non-functional in HEK 293T cells but functional in BMDMs, potentially owing to species-related differences. Overall, the results of in vitro and cell-based mutagenesis were in full agreement with the cryo-EM structure and the expected functional arrangement of NINJ1 filaments at membrane edges.

Finally, we aimed to assess potential dominant-negative effects of the mutants on endogenous NINJ1. We overexpressed mNINJ1 mutants to an intermediate level insufficient to initiate spontaneous cell lysis in GSDMD^tg^ HeLa cells and induced inflammasome activation via DmrB–caspase-4 as before (Fig. 4f and Extended Data Fig. 8f–i). The K45Q, D53A and Q91A mutants had a dominant-negative effect on inflammasome-mediated PMR, without impairing GSDMD activation, which could result from capping or collapsing of endogenous hNINJ1 filaments. Notably, this assay also revealed that the L121F, L121W and T123L mutants, which were dysfunctional in vitro and in overexpression experiments, conferred additional PMR activity compared with controls in the presence of endogenous wild-type protein. Presumably, these mutations weaken the intermolecular interaction in a way that can be tolerated by some of the subunits in a mixed wild-type and mutant filament, but not in every repeating unit of a filament comprising only mutant protein. In summary, mutagenesis confirmed that the formation of NINJ1 filaments in vitro correlates with the ability to induce PMR in both human and mouse cells.

## Discussion

Here we combined insights from super-resolution microscopy, cryo-EM, mutation analysis and molecular dynamics simulation to provide an atomic model for membrane rupture by NINJ1 (Fig. 4g). In live cells, NINJ1 is monomeric in the cellular membrane, with helices α1 and α2 on the extracellular side and the pair of α3 and α4 integral to the membrane (Extended Data Fig. 1a). During cell death, amphipathic helices α1 and α2 insert into the membrane to adopt the kinked conformation, bridging neighbouring protomers to form larger polymers. The resulting higher-polymeric assemblies promote membrane rupture by capping membrane edges, thus stabilizing membrane lesions of variable size and morphology through which LDH, large danger-associated molecular patterns (DAMPs) and other cellular content are released into the extracellular milieu. Although single filaments might be sufficient to damage membranes, it is also plausible that NINJ1 forms double filaments that open up in a zipper-like manner in response to osmotic pressure to form membrane lesions.

**Extended Data Fig. 9:**
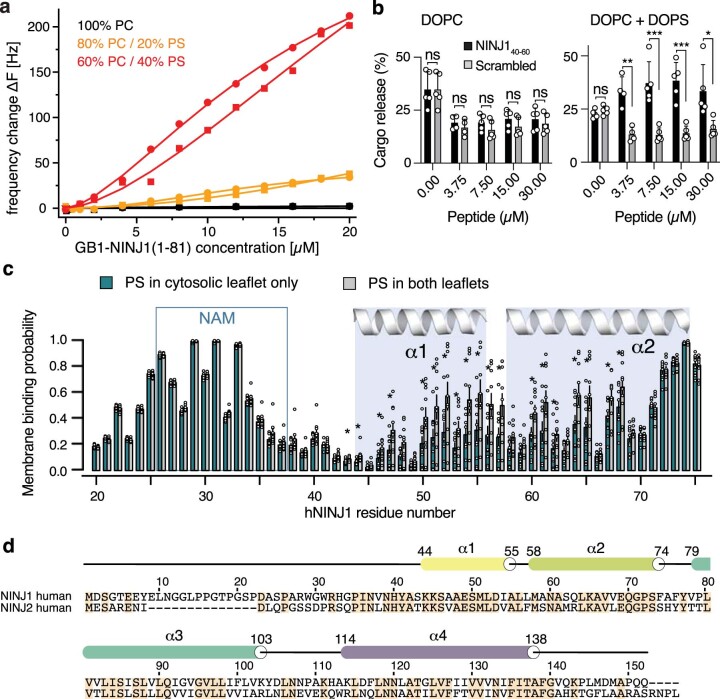
Interactions of the NINJ1 N-terminal helices with membranes. **a**, Quartz crystal microbalance with dissipation (QCM-D) experiments. Frequency change upon addition of GB1-hNINJ1(1–81) to supported lipid bilayer membranes containing only DOPC (black), 80% DOPC/20% DOPS (orange) or 60% DOPC/40% DOPS (red). Duplicate experiments with independently produced protein and lipid bilayers are denoted with squares and circles. The lines denote fits of a Hill equation. **b**, Liposome composition-dependent dye release by hNINJ1_40–60_ peptide or its scrambled sequence version. Data are means of n = 4 replicates for 3.75 µM and n = 5 replicates for the other concentrations, with error bars denoting SD. Significance was calculated using Student’s unpaired two-sided *t*-test. * *P* < 0.05, ** *P* < 0.01, *** *P* < 0.001; ns, not significant. **c**, Residue-specific membrane interaction probability of hNINJ1_20–152_ from CG MD simulations (n = 10). Membrane with PS only in the cytosolic leaflet, corresponding to a healthy cell, are shown in green, while membrane with PS in both leaflets, corresponding to activated cell death signal, is shown as grey bars. The N-terminal adhesion motif (NAM) is highlighted. The error bars denote SEM over the individual simulations (n = 10). Significance was calculated using Student’s paired two-sided *t*-test. * *P* < 0.05, ** *P* < 0.01; *** *P* < 0.001. **d**, Sequence alignment of hNINJ1 and hNINJ2. Conserved residues are highlighted in orange. The secondary structure of hNINJ1 is shown on top. Source data

The trigger that causes the transition of NINJ1 from the inactive state to the active state remains unknown. The proposed polymerization mechanism raises the interesting possibility that the membrane composition might contribute at least partially an activation signal. During cell death, negatively charged phosphatidylserine becomes exposed on the cell surface^28,29^, which might be recognized by helices α1 and α2 of NINJ1. Indeed, lipid binding experiments and dye release assays show that a peptide corresponding to helices α1 and α2 interacts specifically with POPS-containing membranes, and molecular dynamics simulations show the same effect (Extended Data Fig. 9a–c). Membrane-composition sensing as a potential activation mechanism of NINJ1 is thus a promising avenue for future work.

In summary, active NINJ1 has a unique structure with long, α-helical filaments capping membrane edges. Whereas the β-sheet structure of the GSDMD pore has a limited pore size that allows interleukin release while retaining larger molecules^13^, the membrane openings or lesions caused by NINJ1 filaments are essentially unconstrained in size. NINJ1 lesions appear functionally related to the large superstructures of activated mitochondrial Bax and Bak, in that they both lyse membranes^30^. Although atomic structures of Bax or Bak in a pore-forming conformation are not currently available, we speculate that the helical hairpin of α3 and α4 in the NINJ1 filament might show functional and structural resemblance to the helical hairpin of α5 and α6 in activated Bax. NINJ1 was initially reported as an adhesion molecule, induced after sciatic nerve injury and promoting axonal growth^31–33^. Given that NINJ1 drives cell death and the release of DAMPs, and close links exist between cell death, inflammation and tissue repair^34^, it is conceivable that NINJ1 has an indirect role in promoting axonal growth either by causing inflammation or the release of stimulatory molecules. Conversely, it is also possible that NINJ1 has a dual role, serving in both cell–cell adhesion and as a breaking point for membranes at strong osmotic pressure^35^. The structure, along with the mutagenesis studies provide possible explanations for why NINJ2 is not able to functionally replace NINJ1, despite a high degree of homology^8^. The two proteins differ by a few amino acids in the structured part, and by a large deletion and multiple mutations in the unstructured N terminus (Extended Data Fig. 9d). Among the differences in the structured region is residue 47, which is an alanine in NINJ1 and a valine in NINJ2. The A47L mutation made NINJ1 dysfunctional in HEK cells (Fig. 4c), providing a possible explanation for the dysfunction of NINJ2, and it is likely that several of the other differences lead to additional perturbations in function. G93, L57 and Q63 in NINJ1 correspond to V, F and R in NINJ2, respectively, each of which would probably cause steric clashes that might prevent filament formation. Of note, NINJ1 represents yet another occurrence of filament formation in pyroptotic pathways, along with the soluble filaments of PYD and CARD domains of ASC, NLRP3 and caspase-1^30,36–39^. The NINJ1 filament represents an elegant biophysical mechanism of cellular disintegration, and knowledge of its atomic structure opens opportunities for therapeutic interventions in cancer, infection and inflammatory diseases.

## Methods

### Animals

All experiments involving animals were performed under the guidelines of and with approval from the cantonal veterinary office of the canton of Vaud (Switzerland), license number VD3257. All mice were bred and housed at a specific-pathogen-free facility at 22 ± 1 C° room temperature, 55 ± 10% humidity and a day/night cycle of 12h/12h at the University of Lausanne. *Gsdmd*-deficient mice have been described previously^18^. *Ninj1*-deficient mice were generated on the C57BL/6 background at the Center for Transgenic model of the University of Basel as follows: an 8-kb large fragment comprising the entire *Ninj1* locus on mouse chromosome 13 was deleted by CRISPR–Cas9 genome engineering using 2 guide RNAs (gRNAs) (CAGTGCCCGACTCCATGGTGCGG and AGGCCGAGACCCAGTGCGG) binding upstream and downstream of the *Ninj1* locus, respectively. Injection of the gRNAs and Cas9 protein into C57BL/6 embryos was done following published protocols^40^. Biopsies for genotyping were taken at an age of 10–12 days. DNA extraction was performed using the KAPA HotStart Mouse Genotyping Kit according to the manufacturer’s instructions. Genotyping PCR was done using Q5 Polymerase (NEB), carrying out three PCR reactions with three primer sets: PCR-A (GGGCCCGTTTGATCAACAAC and TCGCCGTTGAGCTCATACTC) covering the binding site of gRNA1, PCR-B (GTTCCCTAGCCACTTCCACC and GGCTGGAAGGGTGCTAAGTT) covering the binding site of gRNA2, and PCR-AB (GGGCCCGTTTGATCAACAAC and GGCTGGAAGGGTGCTA AGTT) binding outside of the deleted locus. The expected fragment sizes were 379 bp for PCR-A and 715 bp for PCR-B in animals harbouring a wild-type allele, and 979 bp for PCR-AB in mice harbouring an allele of the deleted locus. The product of PCR-AB was Sanger sequenced to verify the exact extend of the deletion, the deletion was additionally confirmed using anti-mNINJ1 antibodies on cell lysates.

### Plasmids and cloning of NINJ1 mutants for expression in mammalian cells

The plasmid expressing doxycycline (Dox)-inducible C-terminally GFP-tagged hNINJ1 (hNINJ1–GFP) was generated by amplifying the hNINJ1–GFP coding sequence from a commercially obtained plasmid (SinoBiological, HG17094-ACG) by PCR (TACACCGGTGCCGGCGGATCGCCACCATGGACTCGGGAACCGAGGAG; CAGGGGAGGTGG TCTGGATCTTACTTGTACAGCTCGTCCATG CC) and inserting it at the BamHI site of the pLVX-Puro vector (Clontech). cDNA encoding wild-type mNINJ1 was obtained from a commercial plasmid (SinoBiological, MG53796-NF), and cDNAs encoding the different mNINNJ1 mutants were commercially synthesized (GenScript). All mNINJ1 coding sequences were amplified by PCR and subcloned into the BamHI site of the pLVX-Puro vector (Clontech) to generate Dox-inducible plasmids (primers: CACCGGTGCCGGCGGATCGCCACCATGGAGTCGGGCACTGAGGAG; GGGGAGGTGGTCTGGATCTTACTGCTGGGGTGCCATGTC), or into the NotI site of the pMSCV2.2-IRES-GFP vector for retroviral transduction of primary BMDMs (primers: GAGATCGATGCGGCCCGCCACCATGGAGTCGG; CGTTTAAACGCGGCCCTTACTGCCGGGGCG). The plasmid encoding DmrB–Casp4 was generated by amplifying the DmrB^18^ (CAGCCTCGCCACCTCCGCCTT; CCCTCGTAAAGAATTGAGCAAAAGCTC) and the caspase-4 p20/p10 fragment (AGGAGGAGAGGCTGGACCACCTGAGT; GAGGTCGAGAATTCGTCAA TTGCCAGGAAAGAGGTAGAAATATCTTGT) coding sequences, using overlap-extension PCR, and subcloning into the EcoRI site of the pRetroX-Puro vector (Clontech). All cloning was performed using In-Fusion cloning technology (Clontech), and plasmids were verified by sequencing.

### Mammalian cell culture and generation of stable cell lines

Wild-type, *Gsdmd*^–/–^, *Ninj1*^*–/–*^, and *Casp11*^*–/–*^ BMDMs were harvested and differentiated in Dulbecco’s modified Eagle’s medium (DMEM) (Gibco) containing 20% L929 supernatant (as a source of macrophage colony-stimulating factor (MCSF)), 10% heat-inactivated fetal calf serum (FCS) (BioConcept), 10 mM Hepes (BioConcept) penicillin/streptomycin and 1% nonessential amino acids (Gibco), and stimulated on days 8 to 10 of differentiation. Human epithelial HeLa cells (clone CCL-2 from ATCC) and the human embryonic kidney cells HEK 293T were cultured in DMEM containing 10% FCS. All stable cell lines were generated as previously described using a lentiviral transduction protocol^41^. Briefly, 1.5 × 10^6^ HEK 293T cells were transiently transfected with 1.25 µg of the desired lentiviral expression vector together with 250 ng VSVg and 1.25 µg PsPax2, using LT-1 (Mirus) transfection reagent. Next, the lentiviral particles were used to transduce HeLa cells stably expressing Flag–GSDMD–V5 (to generate HeLa expressing Flag–GSDMD–V5 and Dox-inducible DmrB–Casp4)^18^. The stably transduced cell populations were selected using puromycin (5 µg ml^−1^; InvivoGen) for at least 5 days. All cells were grown at 37 °C, 5% CO_2_.

### Retroviral transduction of primary BMDMs

Primary BMDMs were transduced as described previously^42,43^. Genes encoding wild-type or mutant mouse NINJ1 were cloned into pMSCV2.2-IRES-GFP as described above. For transducing primary bone marrow cells of *Ninj1*^–/–^ mice during differentiation to BMDMs, retroviral particles were generated with Phoenix-Eco packaging cells and used to transduce bone marrow cells after 48 h and 72 h of culture in medium with 10% L929-MCSF supernatant (after bone marrow collection). Transduction efficiency was checked by microscopy for GFP expression. Four days after the first transduction, fully differentiated and transduced BMDMs were seeded for experiments.

### Confocal microscopy, time-lapse imaging and image analysis

Wild-type and *Ninj1*^*–/–*^ BMDMs were fixed in 4% PFA for 20 min, washed with PBS, permeabilized with 0.05% saponin and blocked with 1% BSA. Cells were then incubated with an anti-mouse NINJ1 monoclonal antibody (rabbit IgG2b clone 25, Genentech; 1:2,000), Alexa Fluor 488-conjugated anti-rabbit (Life Technologies, 1:500) and Hoechst (1:1,000). For time-lapse imaging of BMDMs, cells were seeded onto 8-well µ-slides (Ibidi), primed overnight with mouse IFNγ and transfected with ultrapure LPS as described below. To visualize phosphatidylserine exposure and membrane permeabilization, the imaging medium (opti-MEM) was supplemented with FITC–annexin V (1 µg ml^−1^; BioLegend) and propidium iodide (5 µg ml^−1^; Thermo Fisher). HeLa cells expressing opto–casp1^14^ were seeded onto 8-well µ-slides and transiently transfected with plasmids encoding Dox-inducible hNINJ1–GFP, which expression was induced for 16 h (1 µg ml^−1^ Dox; Sigma), E-cadherin–GFP or HA^TMD^–GFP. To visualize membrane permeabilization, the imaging medium was supplemented with 1 µM DRAQ7. Samples were then imaged with a Zeiss LSM800 confocal laser scanning microscope using a 63×/1.4 NA oil objective. Time-lapse microscopy was performed at 37 °C with controlled humidity and CO_2_, using a motorized *xyz* stage with autofocus (Zeiss Definite Focus.2 system) and data were acquired using Zeiss ZEN 2 software. Photo-stimulation of opto–casp1 and *z*-stack acquisition (800 nm step size) were performed every 60 s, using the 488-nm laser. Quantification of single-nuclei DRAQ7 intensities was performed using a maximum projection of the *z*-stack, by manually segmenting the nucleus area and measuring the intensity density of the selected regions over time. Fluorescence intensity at each time point (*F*_t_) was then normalized to the intensity at the initial time point of the experiment (*F*_i_)—that is, (*F*_t_ − *F*_i_)/*F*_i_. The distribution pattern of GFP-tagged proteins over time on the plasma membrane of HeLa cells was then assessed using quantitative analysis of the spatial-distributions in images using mosaic segmentation and dual parameter optimization in histograms^44^ (QuASIMoDOH), at the basal plane of cells. The distribution inhomogeneity at each time point (*D*_t_) was normalized to the distribution inhomogeneity at the initial time point of the experiment (*D*_i_)—that is, *D*_t_ − *D*_i_. Time *t* = 0 was defined when DRAQ7 (*F*_t_ – *F*_i_)/*F*_i_ > 1, which corresponds to a significant increase in DRAQ7 nuclear fluorescence intensity and onset of plasma membrane permeabilization. All microscopy datasets were analysed and processed using Fiji software.

### Cell lysis assays in NINJ1 overexpressing cells

HeLa (stably expressing Flag–GSDMD–V5 and DmrB–Casp4) and HEK 293T cells were seeded in 96-well plates at a density of 8.5 × 10^3^ and 1.2 × 10^4^ cells per well, respectively, a day before transfection. The cells were then transiently transfected with 50 ng per well of a pLVX DNA plasmid encoding wild-type or different mNINJ1 mutants, using X-tremeGENE 9 DNA Transfection Reagent (Roche) according to the manufacturer’s instruction. To induce the transgene expression, the medium was supplemented with Dox (1 μg ml^−1^) for 16 h (HeLa) or 48 h (HEK 293T). In HeLa cells, medium was replaced with opti-MEM (Gibco) and cells were incubated with 20 nM B/B homodimerizer (Takara, Clontech) for 1.5 h to activate DmrB–Casp4.

### Inflammasome assays

A day before stimulation, BMDMs were seeded in 96-well plates at a density 5 × 10^4^ per well. To activate canonical inflammasomes cells were first primed with Pam_3_CSK_4_ (1 µg ml^−1^; InvivoGen) for 4 h, washed and then stimulated in opti-MEM with nigericin (5 µg ml^−1^; Sigma-Aldrich), transfected with poly(dA:dT) (13 ng per well; InvivoGen) using Lipofectamine LTX according to the manufacturer’s instructions (Thermo Fisher) or infected with *Salmonella enterica* serovar Typhimurium strain SL1344. Bacteria were grown at 37 °C overnight in an orbital shaker in lysogeny broth (LB) medium containing streptomycin (50 µg ml^−1^). The next day, *Salmonella* cultures were sub-cultured 1/50 and grown until *A*_600_ = 1.5–1.8, collected by centrifugation, washed and resuspended in Opti-MEM. Bacteria were added at a multiplicity of infection (MOI) of 5 for infection of BMDMs, and incubated for 30 min at 37 °C. Next, gentamicin (30 µg ml^−1^; Gibco) was added to kill extracellular bacteria and cells were incubated for the remainder of the experiment. To activate the non-canonical inflammasome, macrophages were first primed overnight with mouse IFNγ (10 ng ml^−1^; PeproTech) in combination with Pam_3_CSK_4_ for 4 h, washed and transfected in Opti-MEM with ultrapure *E. coli* O111:B4 LPS (1.0 µg per 50,000 cells; InvivoGen), using 0.25% FuGENE HD (Promega) as previously described^45^. All treatments and infection times are indicated in figure legends.

### Crosslinking assays

BMDMs were seeded in 24-well plates at a density of 2 × 10^5^ cells per well a day before stimulation, primed and treated with nigericin as described above. After nigericin stimulation (indicated in the figure legends), the crosslinker BS^3^ (bis(sulfosuccinimidyl)suberate) was added to the media (3 mM; Thermo Fisher) and incubated for 5 min at room temperature. Next, a solution of 20 mM Tris pH 7.5 was added to stop the reaction and incubated for 15 min at room temperature. Cell supernatants were collected, proteins were precipitated and combined with cell lysates for western blotting analysis.

### CFSE labelling and co-culture experiments

BMDMs were labelled with CFSE (5 μM; Thermo Fisher) in PBS for 12 min at room temperature in the dark. CFSE was quenched by the addition of assay medium. Then, cells were pelleted, counted, mixed with unlabelled wild-type or *Casp11*^–/–^ BMDMs at a 1:1 ratio and seeded at a density of 2.5 × 10^5^ total cells per well in 24-well plates. Cells were then primed and transfected with ultrapure LPS as described above, stained with propidium iodide (0.5 µg ml^−1^) and imaged on a Cytation 5 imaging plate reader.

### Cell lysis, cell permeabilization and cytokine release

Cell lysis was quantified by measuring the amount of LDH in the cell supernatant using the LDH cytotoxicity kit (Takara, Clontech) according to the manufacturer’s instructions and expressed as a percentage of total LDH release. LDH release was normalized to untreated control and 100% lysis and the percentage of cell lysis was calculated as follows: ([LDH_sample_] – [LDH_negative control_])/([LDH_100% lysis_] – [LDH_negative control_]) × 100%. To measure cell permeabilization, propidium iodide was added to the media (12.5 μg ml^−1^; Thermo Fisher Scientific) and its influx was measured over time using a fluorescence plate reader (Cytation 5; Biotek). A 100% permeabilization control was obtained by adding Triton X-100 to a final concentration of 0.05% and the percentage of propidium iodide uptake was calculated as follows: ([PI_sample_] – [PI_negative control_])/([PI_100% permeable_] – [PI_negative control_]) × 100%. Release of IL-1β into the supernatants was measured by enzyme-linked immunosorbent assay (ELISA) according to the manufacturer’s instructions (Thermo Fisher Scientific).

### Immunoblotting

For western blotting analysis, cells were lysed in 66 mM Tris-HCl pH 7.4, 2% SDS, 10 mM DTT, NuPage LDS sample buffer (Thermo Fisher). When mentioned, supernatants were precipitated with methanol and chloroform, using standard methods, and combined with the cell lysates. Proteins were separated on 4–15% polyacrylamide gels (Bio-Rad) and transferred onto nitrocellulose membrane using Transblot Turbo (Bio-Rad). The antibodies used were: anti-mouse GSDMD (EPR19828; Abcam; 1:1,000), anti-mouse NINJ1 monoclonal antibody (rabbit IgG2b clone 25; a gift from Genentech; 1:8,000), anti-human caspase-4 (ADI-AAM-114-E; Enzo Life Sciences; 1:1,000), anti-V5 (46-0705; Invitrogen; 1:2,000) and mouse anti-tubulin (ab40742; Abcam; 1:2,000). Purified human NINJ1 was detected using mouse anti-human NINJ1 (610777; BD Transduction Laboratories; 1:1,000). Primary antibodies were detected with horseradish peroxidase (HRP)-conjugated goat anti-rabbit (4030-05; Southern Biotech; 1:5,000), HRP-conjugated goat anti-mouse (1034-05; Southern Biotech; 1:5,000 or 12–349; MilliporeSigma; 1:2,000) secondary antibodies.

### STORM sample preparation

Hela cells stably expressing DmrB–Casp4 and GSDMD were seeded in six-well plates on 25 mm glass cover clips (Menzel, #1.5) at a density of 2 × 10^5^ cells per well. The cells were cultivated for 24 h in DMEM (Thermo Fisher) supplemented with 10% fetal calf serum (Thermo Fisher) at 37 °C and 5% CO_2_. The cells were transfected with plasmids encoding hNINJ1–GFP using jetOptimus (Polyplus) transfection reagent according to the manufacturers protocol. Six hours later, NINJ1–GFP and DmrB–Casp4 expression was induced by adding Dox at 1 µg ml^−1^ and the cells were further incubated overnight. Dimerization of Casp4 was induced by adding B/B homodimerizer (Takara) at a final concentration of 10 nM and the cells were incubated for 3 h before proceeding to anti-GFP immunofluorescence staining. The cells were washed twice in PBS and fixed for 15 min in pre-warmed fixation solution consisting of 4% paraformaldehyde (PFA, AlfaAesar) in PBS. The sample was washed again in PBS followed by a permeabilization and blocking incubation with 0.2% bovine serum albumin (BSA) and 0.2% Triton X-100 in PBS for 30 min. The cells were incubated with Alexa Fluor 647-conjugated anti-GFP nanobodies (FluoTag-X4, NanoTag; 1:500 dilution) in a blocking buffer (0.2% BSA in PBS) for 2 h. The sample was finally washed three times for 10 min in PBS. For STORM imaging the glass cover slips were mounted into an AttoFluor (Thermo Fisher) imaging chamber. Unless otherwise indicated, all chemicals were purchased from Sigma.

### STORM imaging and data analysis

A Nikon Ti Eclipse NSTORM microscope equipped with a 642 nm laser (F-04306-113, MPB Communications) was used for STORM imaging to switch molecules to the off state. A 405 nm laser (Cube, Coherent) was used to control the return rate of the fluorophores to the emitting state. A F73-888 quad-band dichroic (Chroma) was used to reflect the laser light on the sample. Emitted light from the sample was collected by the objective lens (Apo TIRF 100×, NA 1.49 Oil, Nikon), passed through the dichroic mirror onto an EMCCD camera (iXon DU-897D, Andor). The width of a square camera pixel corresponds to 160 nm on the sample. Imaging was performed using an established photo-switching buffer made of 50 mM Tris with 10 mM NaCl, supplemented with 10% glucose, 50 mM 2-mercaptoethylamine, 0.5 mg ml^−1^ glucose oxidase and 40 µg ml^−1^ catalase. All chemicals were purchased from Sigma. Typically, 10,000 frames were recorded per field of view with continuous laser exposure at 30 ms exposure time. The microscope was controlled using NIS elements (Nikon), single-molecule localization analysis was performed using Decode^46^. Localizations were further processed using custom MatLab (Mathworks) code. Lateral sample drift was corrected via redundant cross-correlation using Thunderstorm^47,48^. Localizations were clustered using DBSCAN and further processed using MatLab (Mathworks)^49^. Images were rendered and cropped for visualization using Thunderstorm^47^ and Fiji^50^. Plotting and statistical analysis were performed using Prism (GraphPad).

### Protein expression and purification

The plasmid with full-length, wild-type hNINJ1(1–152), was ordered from Genescript in a pET28 vector with an N-terminal 8×His-tag followed by a Tobacco Etch Virus (TEV) cleavage site (ENLYFQGS). All hNINJ1 point mutations were generated by site-directed mutagenesis and verified by DNA sequencing. All NINJ1 constructs were recombinantly expressed in Rosetta2 (DE3) *E. coli*, grown at 37 °C in homemade Terrific Broth (TB) supplemented with additional glucose (24 g l^−1^ yeast extract, 12 g l^−1^ tryptone, 10 g l^−1^ glucose, pH 7.5) and buffered with 10× phosphate buffer (23.1 g l^−1^ KH_2_PO_4_, 125.5 g l^−1^ K_2_HPO_4_) to an absorbance of 3.0 and induced with 0.1 mM IPTG. After induction, the cells were grown for 16 h at 18 °C and cell pellets were harvested and stored at –80 °C. All lysis and purification steps were performed at 4 °C. Cell pellets were resuspended in lysis buffer (50 mM Tris-HCl pH 8.0, 150 mM NaCl), supplemented with lysozyme, DNAse I and Roche Complete protease inhibitor, and lysed with a cell disruptor. The lysate was clarified at 4,400*g* for 35 min. *E. coli* membranes were isolated by ultracentrifugation at 35,000 rpm in a Ti45 rotor. The membranes were gently resuspended in 50 mM Tris-HCl pH 8.0, 150 mM NaCl buffer and manually homogenized with more than 20 strokes. The membrane resuspension was flash frozen in liquid nitrogen and stored at –80 °C. The membrane resuspension was solubilized in 50 mM Tris-HCl pH 8.0, 200 mM NaCl, 15% glycerol, 20 mM imidazole and 1% DDM for >2 h, then centrifuged at 30,000*g* for 30 min. The supernatant was filtered through a 0.45-µm membrane and subsequently loaded onto an equilibrated Ni-NTA column (Cytiva) connected to an Äkta pure system (Cytiva). The column was washed with 10 column volumes of 50 mM imidazole and hNINJ1 was eluted with 5 column volumes containing 400 mM imidazole. The eluate was then desalted using a HiPrep 26/10 desalting column (Cytiva) in a 10 mM Tris-HCl pH 8.0, 150 mM NaCl, 5% glycerol and 0.016% DDM and stored at –80 °C. The 8×His tag was removed by TEV cleavage (16 h at 4 °C with 1:10 molar ratio of TEV). Finally, the sample was loaded on a Superose 6 increase 10/300 column in SEC buffer (10 mM Tris-HCl, pH 8.0, 150 mM NaCl, 5% glycerol and 0.016% DDM) and peak fractions of polymeric hNINJ1 were used for experiments.

For negative-stain transmission electron microscopy (TEM) of hNINJ1 single-point mutants we directly prepared negative-stain TEM samples after TEV cleavage of the 8×His tag. For cryo-EM sample preparation an additional Ni-NTA purification step was included after cleavage of the 8×His tag. The flowthrough was then split into three separate runs of SEC and collected in small fractions in 98-deep well plates. The same high polymeric fraction across collection plates was pooled to yield a concentration appropriate for cryo-EM without the need for additional concentrating steps.

### Protein expression and purification of GB1–NINJ1(1–81)

A wild-type hNINJ1(1–81) construct was purchased from GeneCust in a custom vector designed with a N-terminal 6×His–GB1 tag followed by a TEV cleavage site (ENLYFQG). The construct was recombinantly expressed in *E. coli* BL21(DE3) star cells grown at 37 °C in M9 minimal medium supplemented with 2 g l^−1^ d-[^13^C]-glucose and 1 g l^−1^ [^15^N]-NH_4_Cl, to an absorbance of 0.7 and induced with 1 mM IPTG. After induction cells were grown at 37 °C for 3 h, then harvested and stored at –70 °C. Cell pellet was resuspended in lysis buffer (20 mM Tris-HCl pH 8.0, 300 mM NaCl) supplemented with Roche Complete protease inhibitor, and lysed by ultrasonication. Lysate was centrifuged at 46,000*g* for 1 h at 4 °C and inclusion bodies were dissolved for 16 h at 4 °C in 6 M urea, 20 mM Tris-HCl pH 8.0, 500 mM NaCl. Solubilized inclusion bodies were centrifuged at 46,000*g* for 1 h at 4 °C and supernatant was loaded on Ni^2+^ Sepharose affinity column (Cytiva). The column was washed with 5 column volumes with 20 mM imidazole, then refolded on column with 20 column volumes of refolding buffer (20 mM Tris-HCl pH 8.0, 500 mM NaCl), washed again with 5 column volumes with 20 mM imidazole and recombinant protein was eluted with 5 column volumes of elution buffer (20 mM Tris-HCl pH 7, 100 mM NaCl, 250 mM imidazole). Finally, the sample was loaded on a HiLoad 16/60 Superdex S75 pg column (Cytiva) in SEC buffer (20 mM Tris-HCl pH 7, 100 mM NaCl). Peak fractions of GB1–NINJ1(1–81) were pooled, supplemented with 20 mM sodium cholate and concentrated on an Amicon 4 (MWCO 10 kDa). Typical NMR samples were at a concentration of 50 µM GB1–NINJ1(1–81) (in the absence of detergent) to 500 µM (for the assignment experiments with a sample containing 20 mM sodium cholate). Detergent titrations were performed using individual samples for each detergent concentration (0, 1, 2, 5, 10 and 20 mM cholate).

### Solution NMR spectroscopy

All NMR experiments were performed at 298 K, on a Bruker Avance spectrometer at 800-MHz ^1^H Larmor frequency, equipped with a cryogenic probe. All NMR data were analysed with CCPN (version 3). For the resonance assignment of GB1–NINJ1(1–81), the following experiments were performed: 2D BEST-TROSY, 3D BEST-TROSY HNCO, 3D BEST-TROSY HNcaCO, 3D BEST-TROSY HNCACB, 3D BEST-TROSY HNcoCACB, 3D BEST-TROSY HNcacoNH, 3D BEST-TROSY HNcocaNH^51^. The experiments were performed with a 0.6 mM [*U*-^15^N,^13^C]-labelled sample containing 20 mM sodium cholate and 10% (v/v) D_2_O. ^15^N *R*_2_ spin relaxation measurements were performed on a 50 µM sample of GB1–NINJ1(1–81) without detergent with a CPMG pulse sequence (10 delay values, 24 h total experimental time), and analysed with in-house Python scripts.

### Determination of hNINJ1 polymer mass

For mass photometry measurements the hNINJ1 samples were taken directly after purification and diluted to nanomolar concentrations using SEC buffer. The measurements and data analysis were conducted according to a standard protocol^52^.

### Cryo-EM sample preparation and data collection

For Cryo-EM grids of hNINJ1, the protein concentration was 1.5 mg ml^−1^ in SEC buffer (10 mM Tris-HCl, pH 8.0, 150 mM NaCl, 5% glycerol and 0.016% DDM). A sample volume of 4 µl was applied onto a glow discharged grid (30 s at 50 mA) and blotted for 5 s before being plunge frozen in liquid ethane. Cryo-EM data was collected on a Titan Krios (FEI) mounted with a K2 summit detector (Gatan) using SerialEM 3.8.3^53^. Nominal magnification was 105,000×, corresponding to a pixel size of 0.82 Å per pixel. Movies were collected at 40 frames in multishot and 4 s of total exposure, corresponding to an electron dose of 47 e^−^ Å^−2^.

### Cryo-EM data processing

All movies of the hNINJ1 dataset were imported into CryoSPARC v3.2/v3.3.2^54^. Movies were initially aligned using motion correction and contrast transfer function (CTF) was determined using patch CTF determination. Initial particle picking was done using the filament tracer with filament diameters ranging from 30–60 Å on a subset of micrographs, corresponding particles were then extracted with a box size of 300 pixels. After several rounds of 2D classification, classes showing either side or top view of the filament were chosen as templates for filament tracing on the entire dataset yielding a total of 3,900,041 extracted particles (box size 300 pixels). After additional rounds of 2D classification, 1,085,837 particles were used for ab initio model generation. From inspection of ab initio densities in combination with power spectra analysis of well-resolved 2D classes, we estimated helical parameters of the hNINJ1 filaments. After additional particle clean-up by further 2D classifications and through heterogenous refinement against a lowpass filtered filament density, we were able to conduct a final helical refinement yielding a cryo-EM density that after correction for higher-order aberrations and additional CTF correction per exposure group resulted in a 0.143 gold-standard Fourier shell correlation at 3.8 Å, a rise of 20.95 Å per subunit and a helical twist of −1.05°.

### Model building of hNINJ1 filaments

A single helical subunit repeat was extracted using UCSF ChimeraX^55^ and initial de-novo model building was done using Coot^56^. The initial model building was aided by standard poly-alanine helices and in a later step by an AlphaFold2^19^ structure prediction of hNINJ1. Subsequently the initial model was fitted into three consecutive hNINJ1 protomer densities of one hNINJ1 filament as well as three consecutive hNINJ1 protomer densities on the second, opposing hNINJ1 filament. The map was then zoned around the atomic coordinates using UCSF ChimeraX. The six-protomer map and model were placed in a new unit cell with a P1 space group for subsequent model refinement using default settings in phenix.real_space_refine66^57^ with non-crystallographic symmetry group definitions restraining the helical subunit repeats. After each round of refinement, model geometry was evaluated using MolProbity v4.5.2 Webserver^58^ and problematic or poorly fitting regions were adjusted manually using Coot. This process was repeated until satisfactory levels of model:map agreement and model stereochemistry were achieved (Extended Data Table 1).

### Evolutionary couplings analysis

The computational analysis of evolutionary couplings was performed using the EvCouplings V2 webserver^59^. The query sequence of hNINJ1 (UniprotID: Q92982) spanning residues 33–144 was selected. The default settings were employed, except that the “Sequence fragment filter” option was increased to 80% (from the default value of 50%) in order to remove short fragment sequences from the multiple sequence alignment. The results selected for further analysis were obtained at 0.1 bitscore sequence inclusion threshold. The top 100 pairs with the EV coupling score >0.5 were further analysed based on the hNINJ1 filament structure. Among these, nine coupled amino acid pairs had an intermolecular distance that was shorter than the intramolecular one. Pairs with distances above 12 Å as measured between the Cα atoms were not considered.

### Negative-stain grid preparation and TEM data collection

Five microlitres of 0.02 mg ml^−1^ purified protein were applied to glow discharged carbon-coated copper grids. After 1 min of incubation the grids were rapidly washed in three successive drops of deionized water and exposed to two drops of 2% uranyl acetate solution. Images were recorded on a TEM (CM100, Philips) equipped with a CCD camera with a pixel size of 12 Å per pixel and magnifications ranging from 18,000× to 25,000×.

### Proteoliposome reconstitution for permeability assays

Proteoliposomes were reconstituted as described before^60^. Purified NINJ1 was reconstituted in liposomes made of a 3:1 (wt:wt) lipids mix of POPE (Avanti Polar Lipids) and POPG (Avanti Polar Lipids). Liposomes were prepared by extrusion using 0.4-μm polycarbonate filters. The detergent present during reconstitution was removed by incubation with Bio-Beads SM-2 (Bio-Rad). Proteoliposomes were recovered by ultracentrifugation and resuspended in buffer (20 mM Tris-HCl, pH 8.0, 150 mM NaCl, 2 mM β-mercaptoethanol) to a concentration of 120 (or 240) μM of protein and 20 mg ml^−1^ of lipid, flash frozen in liquid N_2_ and stored at −80 °C until use. NINJ1-mutant proteoliposomes were prepared the same way.

### Proteoliposomes permeability assay

Proteoliposomes were diluted 12.5 times in assay buffer (20 mM HEPES, pH 7.5, 150 mM NaCl), and subjected to freeze–thaw cycles to completely exchange the buffer. Nitrobenzoxadiazole-phosphatodylcholine (NBD-PC) was incorporated in the proteoliposomes by freeze–thaw cycles and extrusion using 0.4 μm polycarbonate filters. The proteoliposomes were then diluted to a lipid concentration of 0.5 mg ml^−1^ with assay buffer, and the fluorescence was recorded using a Jasco FP-6500 spectrofluorometer (excitation, 470 nm; emission 535 nm) at 20 °C. The baseline was measured by 180 s, then 5 mM dithionite was added, and fluorescence was recorded for 500 s until plateau. Finally, 0.5% of Triton X-100 was added to disrupt the proteoliposomes, and fluorescence was recorded for 60 s until plateau. Each sample was measured by triplicate. The permeability activities were calculated as permeability = 100 – 200 × (*F*_dit_ – *F*_triton_)/(*F*_initial_ − *F*_triton_), where *F*_initial_ is the starting fluorescence signal, *F*_dit_ is the plateau value after adding dithionite, and *F*_triton_ is the plateau value after adding Triton X-100.

### Molecular dynamics simulation

An overview of all simulations is given in Supplementary Table 1. The stability of the hNINJ1 polymers was probed by multiscaling molecular dynamics simulations. Coarse-grained molecular dynamics simulations (Martini resolution) were employed over simulation times of multiple tens of µs in multiple replicas. All-atom molecular dynamics simulations using the CHARMM36m force field were performed to complement these long-term simulations with information on adaptation and flexibility of the secondary structure of the N-terminal helix. The same combination of coarse-grained and all-atom methods has proven useful recently for structural adaptability and pore opening of membrane perforating polymers formed by gasdermin A3^26^. The structure of hNINJ1_39–152_ was based on the cryo-EM structure. The C terminus was negatively charged and the capped N terminus was uncharged. In one control simulation, α1 and α2 were combined into a single helix as in the AlphaFold2 model (the helix comprised residues 40–70)^19^. In control simulations lacking the N-terminal helices, hNINJ1_76–152_ was used. In the systems used to study the interactions of the N terminus with the membrane surface, also the N-terminal adhesion motif (NAM) was included, resulting in hNINJ1_20–152_. The plasma membrane model consisted of 1-palmitoyl-2-oleoyl-*sn*-glycero-3-phosphocholine (POPC), cholesterol, 1-palmitoyl-2-oleoyl-*sn*-glycero-3-phosphoethanolamine (POPE) and 1-palmitoyl-2-oleoyl-*sn*-glycero-3-phosphoserine (POPS) in 60:30:5:5 molar ratio and the lipids were symmetrically distributed in the two leaflets, thus mimicking the apoptotic state. In simulations labelled ‘asymmetric’, representing the preapoptotic state, POPE and POPS were asymmetrically distributed between the two leaflets, with POPS located only in the cytosolic and POPE only in the extracellular membrane leaflet. The membrane–protein systems were prepared using the tool insane^61^ and (after equilibration or for visualization purposes) converted back to atomistic resolution using backward^62^.

All simulations were performed at room temperature (293 K) using GROMACS 2020 or 2021^63,64^. In all-atom simulations CHARMM36m^65,66^ was used in combination with the TIP4p water model^67^. Details on simulation setup parameters can be found in our recent publication^68^. In case of ‘endless’ linear polymers, the compressibility of the box along the polymer was set to 4.5 × 10^−7^ bar^−1^. For coarse-grained simulations of hNINJ1 filaments, the polarizable variant of the Martini2 force field was utilized despite its lower computational efficiency^69–71^, as both our initial test simulations on double filaments and literature data show polarizable Martini2 being capable of maintaining fibrous or filament structures^72^. The simulation parameters were taken from de Jong et al., as recommended for GROMACS 5 or newer and polarizable Martini2^73^. We used Martini3 for the estimation of N terminus interactions with the membrane surface, because Martini2 overestimates membrane binding of amphipathic peptides to lipid membranes^74,75^. The simulation parameters can be found in our previous work^26^. Residue-specific membrane interaction probability of hNINJ1_20–152_ was estimated as the probability of each residue to be found in contact (distance of less than 0.63 nm) with the lipids in each simulation (*n* = 10).

### Quartz crystal microbalance with dissipation experiments

Experiments were performed with a QSense Analyzer from Biolin Scientific equipped with three parallel silica-coated sensors (QSX 303). Prior to the experiment, the sensors were cleaned for 30 s in a Zepto plasma cleaner and mounted in the quartz crystal microbalance with dissipation (QCM-D) detection chambers. For the preparation of the supported lipid membrane, small unilamellar vesicles were prepared from mixtures of chloroform stock solution of DOPC (18:1 (Δ9-*cis*) PC; 1,2-dioleoyl-*sn*-glycero-3-phosphocholine) and DOPS (18:1 phosphatidylserine; 1,2-dioleoyl-*sn*-glycero-3-phospho-l-serine) at ratios of 100:0, 80:20 or 60:40 (DOPC:DOPS) for the three series of measurements, respectively, at a concentration of 250 µM. The small unilamellar vesicles were disrupted to form supported bilayers by treatment with 2 mM calcium chloride. After bilayer formation on the sensors, buffer solution was injected and the signal was recorded until a stable frequency signal (set to zero) was reached. Subsequently, solutions of GB1–NINJ1(1–81), expressed in M9 medium and purified as described above, at increasing concentrations were injected, and the time course of the frequency (5th acoustic harmonic) was recorded. Frequency changes, Δ*F*, were obtained as the difference between the plateau value of the frequency before injection and the frequency once equilibrium has been reached after injection^76^. The flow rate in all experiments was 20 µl min^−1^ and the temperature was set to 25 °C. Duplicate experiments were obtained using independent production batches of protein and independently prepared lipid bilayers.

### Liposome preparation

The liposome perforation assay was performed as described following the protocol for GSDMD pore-forming assay^77^. The DOPC and DOPS lipids were purchased from Avanti Polar Lipids. Chloroform lipid solutions at a concentration of 5 mg ml^−1^ were gently dried in a glass tube into a thin film under nitrogen flow and placed under vacuum overnight to further evaporate any residual solvent. To prepare dye-filled liposomes, the dry lipid film was hydrated with 0.5 ml of 50 mM HEPES, 50 mM NaCl, 70 mM 6-carboxyfluorescein, pH 7.5 under shaking at 37 °C for 2 h. The lipid dispersions were subjected to 10 freeze–thaw cycles, and the resultant liposomes were extruded 20 times through 100-nm polycarbonate membranes. The mean size diameter of the liposomes was verified by dynamic light scattering (DLS). The removal of extra-vesicular dye was achieved by a purification step through a PD-10 column (GE Healthcare) pre-equilibrated with 50 mM HEPES, 150 mM NaCl, pH 7.5. All liposomes were stored at 4 °C and used within 24 h.

### Liposome leakage assay

Membrane leakage experiments were performed using 6-carboxyfluorescein containing liposomes composed of 100% DOPC or 80% DOPC and 20% DOPS. The samples were prepared by diluting 10 mM total lipid concentration to 25 µM in 50 mM HEPES buffer, 150 mM NaCl, pH 7.5 supplemented with different concentrations of NINJ1_40–60_ peptide, or its scrambled version (IAAAAMKMYLANSLEHAKSLKV VLASQDSE), or 5% DMSO diluent. The membrane leakage was detected by measuring the end point fluorescence resulting from the dye dilution and subsequent dequenching. The experiments were carried out in a Corning 96-well plate, and the fluorescence was recorded using a Tecan Spark plate reader with excitation and emission wavelengths at 485 and 535 nm, respectively. The percentage of dye release was calculated by normalizing to the emission intensity resulting from the addition of Triton X-100 to the liposome solution.

### Data analysis and statistics

Data analysis was performed using the softwares Gen5, GraphPad Prism v9 and Microsoft Excel. Statistical significance was assessed using Student’s two-tailed *t*-test (for comparison of two groups) or one-way analysis of variance (ANOVA) with Dunnett’s multiple comparisons tests when comparing three or more groups. *P < 0.05, **P < 0.01, ***P < 0.001 and ****P < 0.0001.

### Reporting summary

Further information on research design is available in the Nature Portfolio Reporting Summary linked to this article.

## Online content

Any methods, additional references, Nature Portfolio reporting summaries, source data, extended data, supplementary information, acknowledgements, peer review information; details of author contributions and competing interests; and statements of data and code availability are available at 10.1038/s41586-023-05991-z.

### Supplementary information

Supplementary Information This file contains Supplementary Table 1 (Summary of molecular dynamics simulations), Supplementary Table 2 (Analysis of NINJ1 evolutionary couplings), Supplementary Fig. 1 (Uncropped gels of Fig. 1), Supplementary Fig. 2 (Uncropped gels of Extended Data Fig. 1), Supplementary Fig. 3 (Uncropped gel of Extended Data Fig. 5), Supplementary Fig. 4 (Uncropped gels of Extended Data Fig. 8).

Reporting Summary

Supplementary Video 1 Time-lapse confocal microscopy of primed wild-type BMDMs upon transfection with purified LPS. During pyroptosis of wild-type cells, the plasma membrane blebs (starting at 1 h 35 min, white arrows) collapsed. Pyroptotic cells lost membrane integrity (acquisition of propidium iodide) and were labeled by annexin-V (labels phosphatidylserine exposure to the outer leaflet of the plasma membrane). DIC, differential interference contrast.

Supplementary Video 2 Time-lapse confocal microscopy of primed *Ninj1*^–/–^ BMDMs upon transfection with purified LPS. During pyroptosis of *Ninj1*^–/–^ cells, the plasma membrane blebs (starting at 1 h 45 min, white arrows) inflated without collapsing. Pyroptotic cells lost membrane integrity (acquisition of propidium iodide) and were labeled by annexin-V (labels phosphatidylserine exposure to the outer leaflet of the plasma membrane). DIC, differential interference contrast.

Supplementary Video 3 Time-lapse fluorescence confocal microscopy of pyroptotic HeLa cells expressing hNINJ1–GFP. HeLa cells co-expressing hNINJ1–GFP (green) and opto-Casp1 were photo-activated with 488 nm light to trigger caspase-1-induced pyroptosis. Loss of plasma membrane integrity was measured by influx of DRAQ7 (purple). DRAQ7 images show a maximum projection from a *z*-stack, and hNINJ1–GFP images show the basal or central plane of the cell. Time was normalized to the onset of increase in DRAQ7 nuclear fluorescence.

Supplementary Video 4 Time-lapse fluorescence confocal microscopy of pyroptotic HeLa cells expressing HA^TMD^–GFP. HeLa cells co-expressing HA^TMD^–GFP (green) and opto–CASP1 were photo-activated with 488-nm light to trigger caspase-1-induced pyroptosis. Loss of plasma membrane integrity was measured by influx of DRAQ7 (purple). DRAQ7 images show a maximum projection from a *z*-stack, and HA^TMD^–GFP images show the basal or central plane of the cell. Time was normalized to the onset of increase in DRAQ7 nuclear fluorescence.

Supplementary Video 5 Time-lapse fluorescence confocal microscopy of pyroptotic HeLa cells expressing E-cadherin–GFP. HeLa cells co-expressing E-cadherin–GFP (green) and opto–CASP1 were photo-activated with 488 nm light to trigger caspase-1-induced pyroptosis. Loss of plasma membrane integrity was measured by influx of DRAQ7 (purple). DRAQ7 images show a maximum projection from a *z*-stack, and E-cadherin–GFP images show the basal or central plane of the cell. Time was normalized to the onset of increase in DRAQ7 nuclear fluorescence.

Supplementary Video 6 Time evolution of a 45-ring hNINJ1_39–152_ in the course of a coarse-grained simulation. The shape of a ring comprising 45 hNINJ1_39–152_ fluctuates over the course of a 150-µs-long coarse-grained simulation, yet the ring stays stable and open. Lipids are shown as grey sticks with phosphates highlighted as spheres. Protein backbone is shown as thick ribbon and individual chains are randomly coloured in purple, green, or yellow colours.

Supplementary Video 7 Collapse of a 45-ring hNINJ1_79–152_ in the course of a coarse-grained simulation. The collapse of a ring consisting of 45 hNINJ1_79–152_ (that is, hNINJ1 missing the NTH part) is followed over a 150-µs-long molecular dynamics simulation at coarse-grained resolution. Lipids are shown as grey sticks with phosphates highlighted as spheres. Protein backbone is shown as thick ribbon and individual chains are randomly colored in purple, green, or yellow colours.

### Source data

Source Data Figs. 1–4 and Source Data Extended Data Figs. 1, 3, 4, 6–9.

## Figures and Tables

**Extended Data Table 1:**
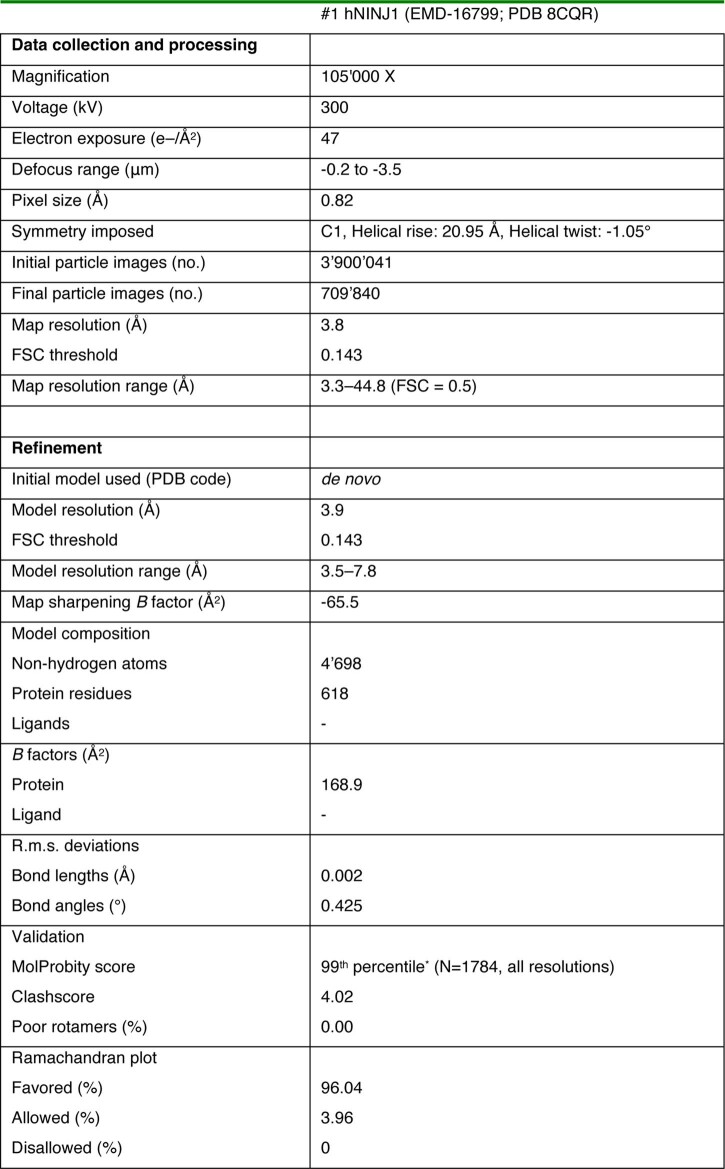
Cryo-EM data collection, refinement and validation statistics

## Data Availability

The atomic coordinates of filamentous hNINJ1 have been deposited in the RCSB Protein Data Bank with the accession code 8CQR. The cryo-EM map has been deposited in the Electron Microscopy Data Bank (EMDB) with accession code EMD-16799. All other data that support the findings of this study are available from the corresponding authors upon request. Source data are provided with this paper.

## Code availability

Data and scripts used to analyse the molecular dynamics simulations have been deposited in the DaRus repository under the link 10.18419/darus-3373. Code used for super-resolution data processing are available at https://github.com/christian-7/. 
