# Supplementary material for: Structural basis of NINJ1-mediated plasma membrane rupture in cell death
Source: Nature. 2023 May 17;618(7967):1065–71. doi: 10.1038/s41586-023-05991-z (PMC10307626; doi:10.1038/s41586-023-05991-z)
Supplement: Supplementary file 1 — This file contains Supplementary Table 1 (Summary of molecular dynamics simulations), Supplementary Table 2 (Analysis of NINJ1 evolutionary couplings), Supplementary Fig. 1 (Uncropped gels of Fig. 1), Supplementary Fig. 2 (Uncropped gels of Extended Data Fig. 1), Supplementary Fig. 3 (Uncropped gel of Extended Data Fig. 5), Supplementary Fig. 4 (Uncropped gels of Extended Data Fig. 8). [file 41586_2023_5991_MOESM1_ESM.pdf]

nature portfolio

https://doi.org/10.1038/s41586-023-05991-z

Supplementary information

Structural basis of NINJ1-mediated plasma membrane rupture in cell death

In the format provided by the authors and unedited

Supplementary Table 1. Summary of MD simulations

Simulation system	Resolution	Simulation times
Stability of filaments & rings		
Double filament 2x10 hNINJ1$_{39-152}$	CG	50µs, 50µs
Double filament 2x10 hNINJ1$_{39-152}$, with DDM	CG	50µs, 50µs
Single filaments at membrane patch 2x10 hNINJ1$_{39-152}$	CG	20µs, 20µs
Single filaments at membrane patch 2x5 hNINJ1$_{39-152}$	AA	1µs, 1µs
Single filament in solution, restrained helices α3 & α4, Alphafold2 shape of α1+α2 hNINJ1$_{39-152}$	AA	1µs
45-er ring hNINJ1$_{39-152}$	CG	150µs, 150µs, 150µs, 150µs
45-er ring hNINJ1$_{76-152}$ (helices α3 & α4 only)	CG	150µs, 150µs, 150µs
Membrane binding of the N-terminus		
Monomeric hNINJ1$_{20-152}$, asymmetric membrane	CG	10x40µs
Monomeric hNINJ1$_{20-152}$, symmetric membrane	CG	10x40µs

Supplementary Table 2. Analysis of NINJ1 evolutionary couplings.[a]

Pair number	Residue 1	Residue 2	Probability	Score	$d_{C\alpha\text{-}C\alpha}$ inter [Å]	$d_{C\alpha\text{-}C\alpha}$ intra [Å]
1	G95	F127	98.7	4.35	8.5	12.4
2	S46	V102	91.6	2.38	11.9	14.9
3	K44	G95	77.4	1.23	8.0	16.5
4	N107	H112	75.0	1.1	9.8	10.8
5	M51	F127	68.2	0.76	6.1	20.2
6	I54	G124	66.7	0.7	11.1	16.8
7	S43	V102	64.9	0.62	7.5	17.5
8	I92	I128	63.8	0.56	8.5	12.9
9	K103	N120	62.5	0.51	8.8	17.6

[a]All evolutionary couplings among the 100 with highest score that have a closer spatial proximity for intermolecular distance than the intramolecular distance. See Methods for details

Fig. 1a

Order of antibody incubation (all in the same membrane):
1) anti-NINJ1
2) anti-GSDMD
3) anti-Tubulin

Fig. 1b

Order of antibody incubation (all in the same membrane):
1) anti-NINJ1
2) anti-GSDMD
3) anti-Tubulin

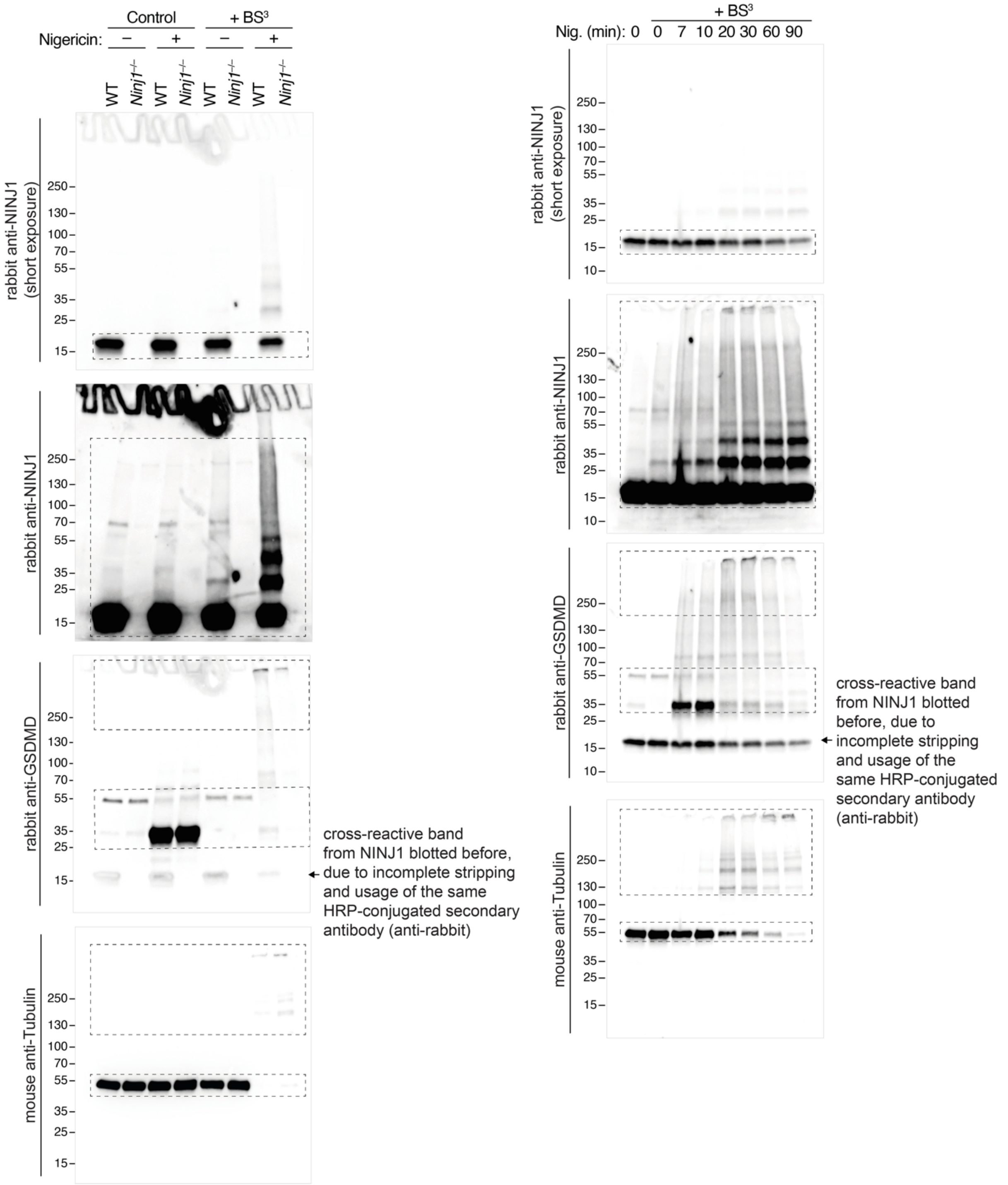

Supplementary Figure 1. Uncropped gels of Figure 1.

ED 1c

Order of antibody incubation
(all in the same membrane):
1) anti-NINJ1
2) anti-Tubulin

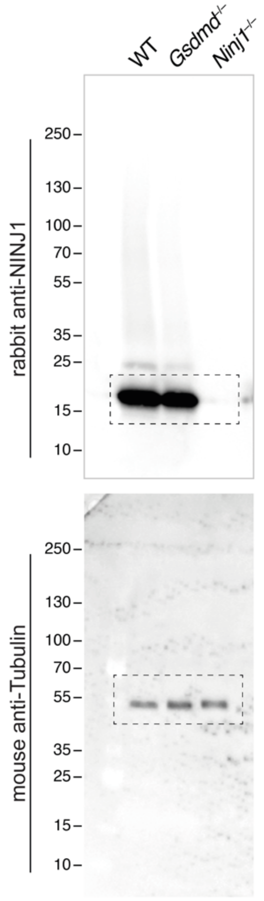

ED 1e

Order of antibody incubation (all in the same membrane):
1) anti-NINJ1 (not shown. Done to validate knock-out cells)
2) anti-GSDMD
3) anti-Tubulin

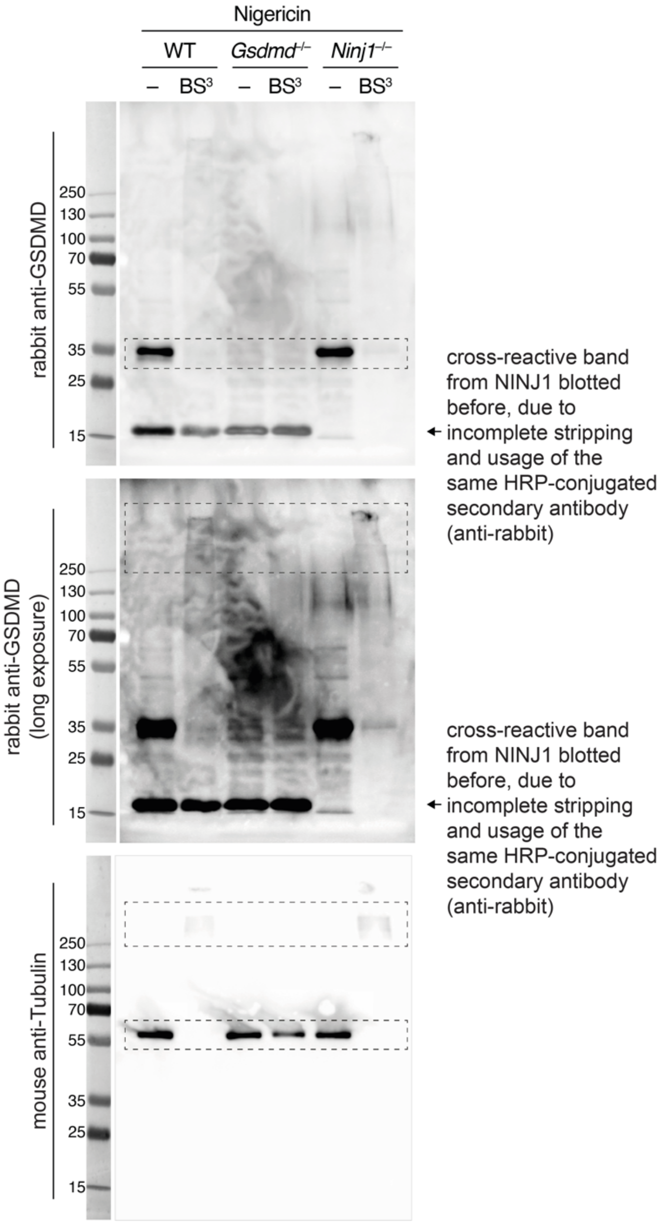

Supplementary Figure 2. Uncropped gels of Extended Data Figure 1.

ED 5c

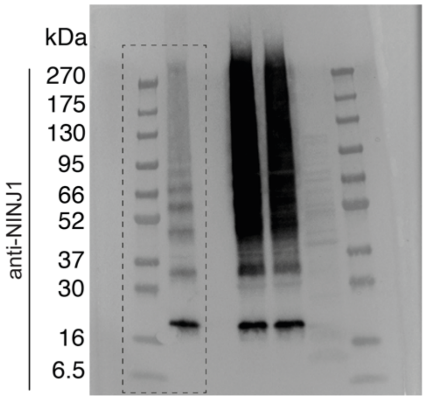

Supplementary Figure 3. Uncropped gel of Extended Data Figure 5.

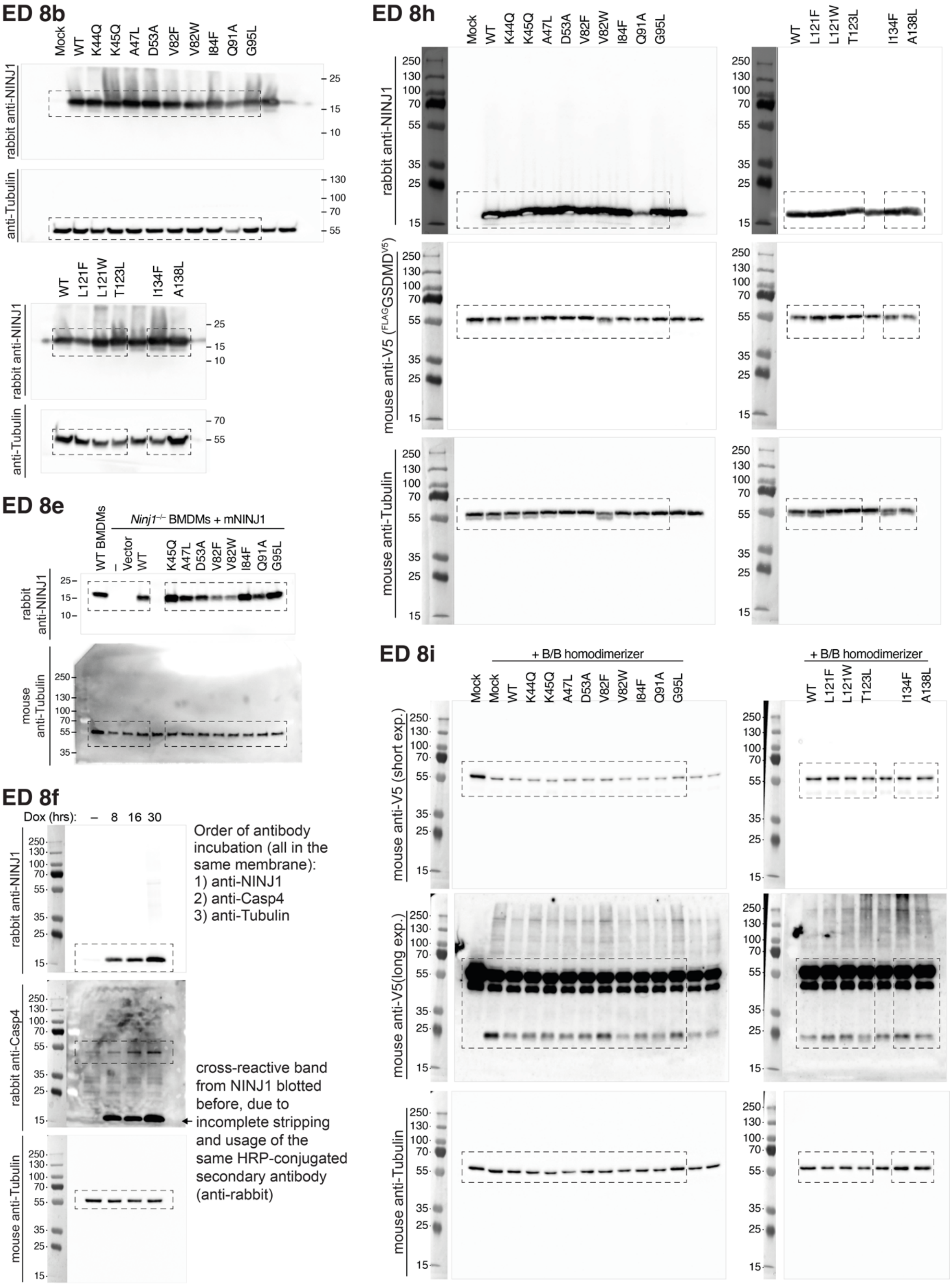

Supplementary Figure 4. Uncropped gels of Extended Data Figure 8.
