# Supplementary material for: Structural basis of NINJ1-mediated plasma membrane rupture in cell death
Source: Nature. 2023 May 17;618(7967):1065–71. doi: 10.1038/s41586-023-05991-z (PMC10307626; doi:10.1038/s41586-023-05991-z)
Supplement: Supplementary file 2 — Reporting Summary [file 41586_2023_5991_MOESM2_ESM.pdf]

nature portfolio

Corresponding author(s): Sebastian Hiller, Petr Broz, Kristyna Pluhackova

Last updated by author(s): Mar 7, 2023

Reporting Summary

Nature Portfolio wishes to improve the reproducibility of the work that we publish. This form provides structure for consistency and transparency in reporting. For further information on Nature Portfolio policies, see our Editorial Policies and the Editorial Policy Checklist.

Statistics

For all statistical analyses, confirm that the following items are present in the figure legend, table legend, main text, or Methods section.

n/a	Confirmed	
☐	☒	The exact sample size (*n*) for each experimental group/condition, given as a discrete number and unit of measurement
☐	☒	A statement on whether measurements were taken from distinct samples or whether the same sample was measured repeatedly
☐	☒	The statistical test(s) used AND whether they are one- or two-sided<br>*Only common tests should be described solely by name; describe more complex techniques in the Methods section.*
☒	☐	A description of all covariates tested
☒	☐	A description of any assumptions or corrections, such as tests of normality and adjustment for multiple comparisons
☐	☒	A full description of the statistical parameters including central tendency (e.g. means) or other basic estimates (e.g. regression coefficient) AND variation (e.g. standard deviation) or associated estimates of uncertainty (e.g. confidence intervals)
☐	☒	For null hypothesis testing, the test statistic (e.g. *F*, *t*, *r*) with confidence intervals, effect sizes, degrees of freedom and *P* value noted<br>*Give P values as exact values whenever suitable.*
☒	☐	For Bayesian analysis, information on the choice of priors and Markov chain Monte Carlo settings
☒	☐	For hierarchical and complex designs, identification of the appropriate level for tests and full reporting of outcomes
☒	☐	Estimates of effect sizes (e.g. Cohen's *d*, Pearson's *r*), indicating how they were calculated

Our web collection on statistics for biologists contains articles on many of the points above.

Software and code

Policy information about availability of computer code

Data collection	NMR: Topspin version 4.1.3 (Bruker Biospin); MD: GROMACS 2020/2021; STORM: Nikon NIS elements version 5.30; Cell biology: Fusion Solo S, Gen5, Zen Blue 2.3 Imaging Software; Negative Stain TEM: iTEM Build 5.2, Cryo-EM: Titan Krios electron microscope (FEI) with GIF Quantum LS Imaging filter (Gatan) and K2 summit (Gatan) operated using SerialEM 3.8.3; SparkControl v2.3
Data analysis	NMR: CCPN version 3.0.4, xmgrace version Grace 5.1.25; MD: GROMACS 2020/2021, R version 3.6.3, PyMol 2.5.0; STORM: Data analysis: Decode v0.1, Matlab 2020b, Fiji with ImageJ 1.53q, ThunderSTORM v1.3, own custom processing codes (https://github.com/christian-7/); Cell biology: Microsoft Excel for Mac v16, Prism Graphpad 9.0, Gen5, FiJi, Zen Blue 2.3 Imaging Software; Structural Biology: Phenix Version 1.19.2-4158; Coot 0.8.9.2 EL, 0.9.7 EL; cryoSPARC v3.2, v3.3.2; ChimeraX v1.2.5,1.3, Alphafold 2.2.0, EvCouplings V2 webserver, MolProbity v4.5.2 Webserver

For manuscripts utilizing custom algorithms or software that are central to the research but not yet described in published literature, software must be made available to editors and reviewers. We strongly encourage code deposition in a community repository (e.g. GitHub). See the Nature Portfolio guidelines for submitting code & software for further information.

Data

Policy information about availability of data

All manuscripts must include a data availability statement. This statement should provide the following information, where applicable:

- Accession codes, unique identifiers, or web links for publicly available datasets
- A description of any restrictions on data availability
- For clinical datasets or third party data, please ensure that the statement adheres to our policy

The atomic coordinates of filamentous hNINJ1 have been deposited in the RCSB Protein Data Bank with the accession code 8CQR. The cryo-EM map has been deposited in the EMDB with accession code EDB-16799. Source data are provided with this paper. All other data that support the findings of this study are available from the corresponding authors upon request. Explanation: This combination of Data Bank Deposition / Source Data Provided / Data upon request is the most efficient combination of data forsharing in terms of curation recources spent vs. user access frequency. Protein sequences were retrieved from the Uniprot database: Q92982 - hNINJ1, O70131 - mNINJ1, Q9NZG7 - hNINJ2.

Human research participants

Policy information about studies involving human research participants and Sex and Gender in Research.

Reporting on sex and gender	NA
Population characteristics	NA
Recruitment	NA
Ethics oversight	NA

Note that full information on the approval of the study protocol must also be provided in the manuscript.

Field-specific reporting

Please select the one below that is the best fit for your research. If you are not sure, read the appropriate sections before making your selection.

☒ Life sciences ☐ Behavioural & social sciences ☐ Ecological, evolutionary & environmental sciences

For a reference copy of the document with all sections, see nature.com/documents/nr-reporting-summary-flat.pdf

Life sciences study design

All studies must disclose on these points even when the disclosure is negative.

Sample size	No statistical method was used to determine sample sizes and no sample size calculation was performed. When using primary mouse bone marrow-derived macrophages, cells from at least 2 animals per genotype were used for reproducibility. Sample sizes were chosen to match our previous published work in which similar experimental setups were used (Meunier et al., Nature 2014; Ruhl et al, Science 2018; Santos et al., Nat Commun 2020). This sample size is also widely used and accepted in the field (Shi et al, Nature 2015; Kayagaki et al, Nature 2015; Kayagaki et al, Nature 2021), yielding statistically significant and publication quality results.
Data exclusions	No data were excluded from the analysis.
Replication	Each experiment was repeated as described in the figure legends. Each replicable experiment was repeated at least three times independently. The number of replicate performed for each figure is stated in the figure legends. Experiments were repeated, when possible, by different experimenters to ensure the reproducibility.
Randomization	There was no randomization for these experiments. This study is not a randomised control trial and randomisation is not conventionally used in in vitro/in cellulo studies such as this one. All groups of experiments were performed using the same experimental conditions and protocols.
Blinding	Assays used objective quantification methods that are not susceptible to bias, so samples were not blinded.

Reporting for specific materials, systems and methods

We require information from authors about some types of materials, experimental systems and methods used in many studies. Here, indicate whether each material, system or method listed is relevant to your study. If you are not sure if a list item applies to your research, read the appropriate section before selecting a response.

Materials & experimental systems

n/a	Involved in the study
☐	☒ Antibodies
☐	☒ Eukaryotic cell lines
☒	☐ Palaeontology and archaeology
☐	☒ Animals and other organisms
☒	☐ Clinical data
☒	☐ Dual use research of concern

Methods

n/a	Involved in the study
☒	☐ ChIP-seq
☒	☐ Flow cytometry
☒	☐ MRI-based neuroimaging

Antibodies

Antibodies used

Antibodies used for western blot:

Mouse monoclonal anti caspase-4 clone 4B9 (Enzo life Sciences, cat #ADI-AAH-114-5; dilution 1:1000)
Mouse monoclonal anti-V5 (ThermoFischer Scientific, cat# R960-25; dilution 1:2000)
Rabbit monoclonal anti-mouse NINJ1 (rabbit IgG2b clone 25, a kind gift from Genentech, no catalogue number; dilution 1:8000)
Rabbit monoclonal anti-GSDMD (EPR19828, Abcam, cat# ab209845; dilution 1:1000)
Mouse monoclonal anti-Tubulin clone DM1A (Abcam, cat# ab40742; dilution 1:2000)
Goat anti-Rabbit IgG-HRP (SouthernBiotech, cat# 4030-05; dilution 1:5000)
Goat anti-Mouse IgG(H+L), Rat ads-HRP (SouthernBiotech, cat#1034-05; dilution 1:5000)
Mouse anti-human NINJ1 (BD Transduction Laboratories cat#610777, lot#1070002; dilution 1:1000)
Goat anti-mouse IgG HRP conjugate (MilliporeSigma cat#12-349, lot#3722026; dilution 1:2000)

Antibodies used for immunofluorescence and STORM:
Rabbit monoclonal anti-mouse NINJ1 (rabbit IgG2b clone 25, a kind gift from Genentech, no catalogue number; dilution 1:2000)
Alexa Fluor 488-conjugated Goat anti-rabbit IgG (H+L) (Life Technologies, cat# A-11008; dilution 1:500)
FluoTag-X4 anti-GFP nanobody (Nanotag, cat# N0304, dilution 1:500)

Validation

Anti-Caspase-4, anti-GSDMD and anti-NINJ1 antibodies were validated using knockout cell lines, validated by the suppliers and are extensively used in the scientific community.
Anti-V5 was validated by overexpressing protein tagged V5-epitope and have been validated by their respective manufacturers. Anti-tubulin has been validated by their manufacturer and is extensively used in the scientific community.

Eukaryotic cell lines

Policy information about cell lines and Sex and Gender in Research

Cell line source(s)

HeLa clone CCL-2 (obtained from ATCC).
HEK293T (ATCC)
Mouse Bone Marrow-Derived Macrophages (BMDMs): primary cells (from both male and female mice)

Authentication

Cell lines were obtained from ATCC and authenticated by the vendor. The identity of cell lines was frequently checked by their morphological features and did not show any signs of cross-contamination.

Mycoplasma contamination

Cell lines are regularly tested in the lab for mycoplasma contamination and are mycoplasma free.

Commonly misidentified lines
(See ICLAC register)

No commonly misidentified cell lines were used in this study.

Animals and other research organisms

Policy information about studies involving animals; ARRIVE guidelines recommended for reporting animal research, and Sex and Gender in Research

Laboratory animals

C57BL/6 mice were used in this study to generate bone marrow-derived macrophages. The specific strains (WT, Ninj1-KO and Gsdmd-KO) are indicated, and BMDMs were harvested from both male and female 8-10 week old mice. All mice were bred and housed at a specific-pathogen-free facility at 22 +/- 1 C° room temperature, 55 +/- 10 % humidity and a day/night cycle of 12h/12h at the University of Lausanne.

Wild animals

This study did not involve wild animals

Reporting on sex

The findings apply to both females and males. No differences have been observed between BMDMs from male or female mice

Field-collected samples

Study did not involve samples collected from the field

Ethics oversight

All experiments involving animals were performed under the guidelines and approval from the cantonal veterinary office of the Canton of Vaud (Switzerland). License number VD3257

Note that full information on the approval of the study protocol must also be provided in the manuscript.
